# Piezo1 Activation Improves NSCLC Liver Metastasis Immunotherapy by Overriding Matrix Stiffness‐Mediated Bimodal PD‐L1/CXCL10 Regulation

**DOI:** 10.1002/advs.202501335

**Published:** 2025-06-29

**Authors:** Tian Zhang, Yuan Li, Bo Cheng, Zhao Xu, Mengjie Liu, Jinteng Feng, Yixue Bai, Yang Yu, Panpan Jiang, Luying Geng, Feng Xu, Hui Guo

**Affiliations:** ^1^ Department of Medical Oncology The Second Affiliated Hospital of Xi'an Jiaotong University Xi'an 710004 P. R. China; ^2^ The Key Laboratory of Biomedical Information Engineering of Ministry of Education Xi'an Jiaotong University Xi'an 710049 P. R. China; ^3^ Bioinspired Engineering and Biomechanics Center (BEBC) Xi'an Jiaotong University Xi'an 710049 P. R. China; ^4^ Phase I Clinical Trial Ward The Second Affiliated Hospital of Xi'an Jiaotong University Xi'an 710049 P. R. China; ^5^ Department of Thoracic Surgery The First Affiliated Hospital of Xi'an Jiaotong University Xi'an 710061 P. R. China; ^6^ The Key Laboratory of Surgical Critical Care and Life Support of Ministry of Education Xi'an Jiaotong University Xi'an 710049 P. R. China

**Keywords:** mechanomedicine, NSCLC liver metastasis, organ‐specific heterogeneity, tumor immune microenvironment, tumor physical microenvironment

## Abstract

Immunotherapy efficacy in NSCLC is significantly reduced upon liver metastasis due to profound alterations in the tumor microenvironment, characterized by the absence of cyclic mechanical stretch and increased extracellular matrix (ECM) stiffness. However, the mechanisms underlying the synergistic regulation of these two mechanical cues on the immunotherapy response in NSCLC cells remain poorly understood. In this study, it is demonstrated that both mechanical and biochemical activation of the mechanosensitive ion channel Piezo 1 induces nuclear translocation of YAP, thereby promoting an immunotherapy‐responsive tumor immune microenvironment (TIME) through enhanced expression of PD‐L1 and secretion of chemokine C‐X‐C ligand 10 (CXCL10, chemokine recruiting CD8^+^ T cells) in NSCLC cells. The mathematical modeling further reveals that cyclic stretch modulates the PD‐L1/CXCL10 response to ECM stiffness, shifting from a bimodal to a unimodal distribution. In a murine model of liver metastasis, the combination of Piezo 1 agonist with anti‐PD‐1 therapy significantly improves the immunotherapy response, as evidenced by elevated PD‐L1 levels and increased CD8^+^ T cell infiltration. These findings underscore the critical role of Piezo 1 in enhancing the immunotherapeutic response in NSCLC liver metastasis and highlight its potential as a therapeutic target.

## Graphical abstract

This study demonstrates that Piezo1 activation enhances FAK‐YAP‐mediated upregulation of PD‐L1 and CXCL10, thereby promoting an immunotherapy‐responsive tumor microenvironment in NSCLC. Cyclic stretch modulates the PD‐L1/CXCL10 response to ECM stiffness, reshaping their expression dynamics. In vivo, Piezo1 activation combined with anti‐PD‐1 therapy significantly enhances immunotherapy efficacy in NSCLC liver metastasis.

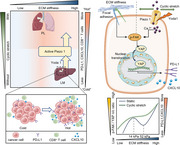

## 1 Introduction

Lung cancer represents a global health challenge for its alarmingly high morbidity and mortality. Non‐small cell lung cancer (NSCLC), which comprises ≈85% of all lung cancer cases^[^ ^1^ ^]^, has experienced a transformation in treatment strategy with the advent of anti‐PD‐1/PD‐L1 immunotherapy, demonstrating exceptional efficacy in select patient populations.^[^ ^2^ ^]^ However, the challenge of metastasis^[^ ^3^ ^]^—particularly to the liver, which affects 15–20% of patients with metastatic NSCLC—severely diminishes the efficacy of anti‐PD‐1/PD‐L1 immunotherapy.^[^ ^4^, ^5^ ^]^ The liver metastasis microenvironment is marked by a tumor immune microenvironment (TIME) largely unresponsive to immunotherapy,^[^ ^6^, ^7^ ^]^ a clinical observation that may be attributed to distinct organ‐specific microenvironmental factors. NSCLC tumors within liver metastases typically exhibit “cold” tumor characteristics,^[^ ^8^ ^]^ indicated by a lack of CD8^+^ T cell infiltration^[^ ^9^ ^]^ and low immunogenicity of tumor cells,^[^ ^10^ ^]^ both of which are detrimental to successful immunotherapy interventions.^[^ ^11^ ^]^ Strategies aimed at converting these “cold” tumors into “hot” ones, characterized by increased immunogenicity and CD8^+^ T cell presence, have shown promise in enhancing immunotherapy efficacy.^[^ ^12^ ^]^ While biochemical interventions, such as indoleamine 2,3‐dioxygenase (IDO) inhibitors,^[^ ^13^ ^]^ have been explored in clinical trials, their limited success suggests that additional regulatory mechanisms may be critical to overcoming immunotherapy resistance.

The tumor physical microenvironment (TPME) is increasingly recognized for its essential role in tumor initiation,^[^ ^14^ ^]^ progression,^[^ ^15^ ^]^ and therapeutic response.^[^ ^16^ ^]^ Emerging evidence underscores its significant impact on TIME regulation.^[^ ^17^ ^]^ For instance, within lung tumors, increased collagen deposition has been associated with CD8^+^ T cell exhaustion, mediated by the suppression of SHP‐1 signaling through interactions between the collagen‐receptor LAIR1 and CD18.^[^ ^18^ ^]^ Moreover, extracellular matrix (ECM) stiffening has been shown to elevate PD‐L1 expression in NSCLC cells via an actin‐dependent pathway.^[^ ^19^ ^]^ The mechanosensitive Ca^2+^ ion channel Piezo1, which is integral to pulmonary innate immune responses, modulates the secretion of C‐X‐C ligand (CXCL) chemokines.^[^ ^20^ ^]^ Notably, Piezo1 can influence the balance between regulatory T (Treg) cells and proinflammatory T helper 1 (Th1) cells, suggesting its potential role in shaping TIME of tumors.^[^ ^21^, ^22^ ^]^ YAP, a well‐known effector of both ECM stiffness^[^ ^23^ ^]^ and cyclic stretch,^[^ ^24^ ^]^ further modulates PD‐L1 expression,^[^ ^25^ ^]^ positioning it as a central player in the mechanotransduction pathways that influence TIME. In the context of liver metastasis, NSCLC cells encounter a distinct TPME characterized by the loss of cyclic stretch and increased ECM stiffness, primarily due to the organ‐specific heterogeneity between the lung and liver. While the significance of ECM stiffness in TIME modulation has been acknowledged in NSCLC cells,^[^ ^19^ ^]^ the impact of cyclic stretch and its synergistic impact with ECM stiffness on TIME remains elusive.

In this study, we unravel that organ‐specific TPME critically regulates the low‐responsive TIME of NSCLC cells at the liver metastatic sites. Our findings demonstrate that the mechanical and biochemical activation of Piezo1 enhances PD‐L1 expression and CXCL10 secretion via the Piezo1‐YAP axis, thereby promoting a more immunotherapy‐responsive TIME. Notably, while ECM stiffness induces a bimodal pattern change of PD‐L1 and CXCL10 expression, the application of cyclic stretch shifts this response to a unimodal curve. This transition is accompanied by consistent changes in the nuclear‐to‐cytoplasmic (n/c) ratio of YAP and the levels of phosphorylated Tyr397 focal adhesion kinase (FAK). With a modified motor‐clutch model, we elucidate that cyclic stretch can reinforce the integrin‐matrix bond at an optimum stiffness threshold of ≈14 kPa, resulting in distinct mechanotransduction outcomes. Furthermore, in a murine model, we demonstrate that Piezo1 activation, in combination with anti‐PD‐1 therapy, effectively transforms the TIME of liver metastatic site from “cold” to “hot” status, significantly enhancing the immunotherapy efficacy. These findings highlight the critical role of TPME in regulating immunotherapy response through TIME and underscore the therapeutic potential of Piezo1 activation in NSCLC treatment, particularly in the context of liver metastasis.

## 2 Results

### 2.1 Both TIME and TPME Variances Correlate with Poor Immunotherapy Response of NSCLC Liver Metastasis

**Figure 1:**
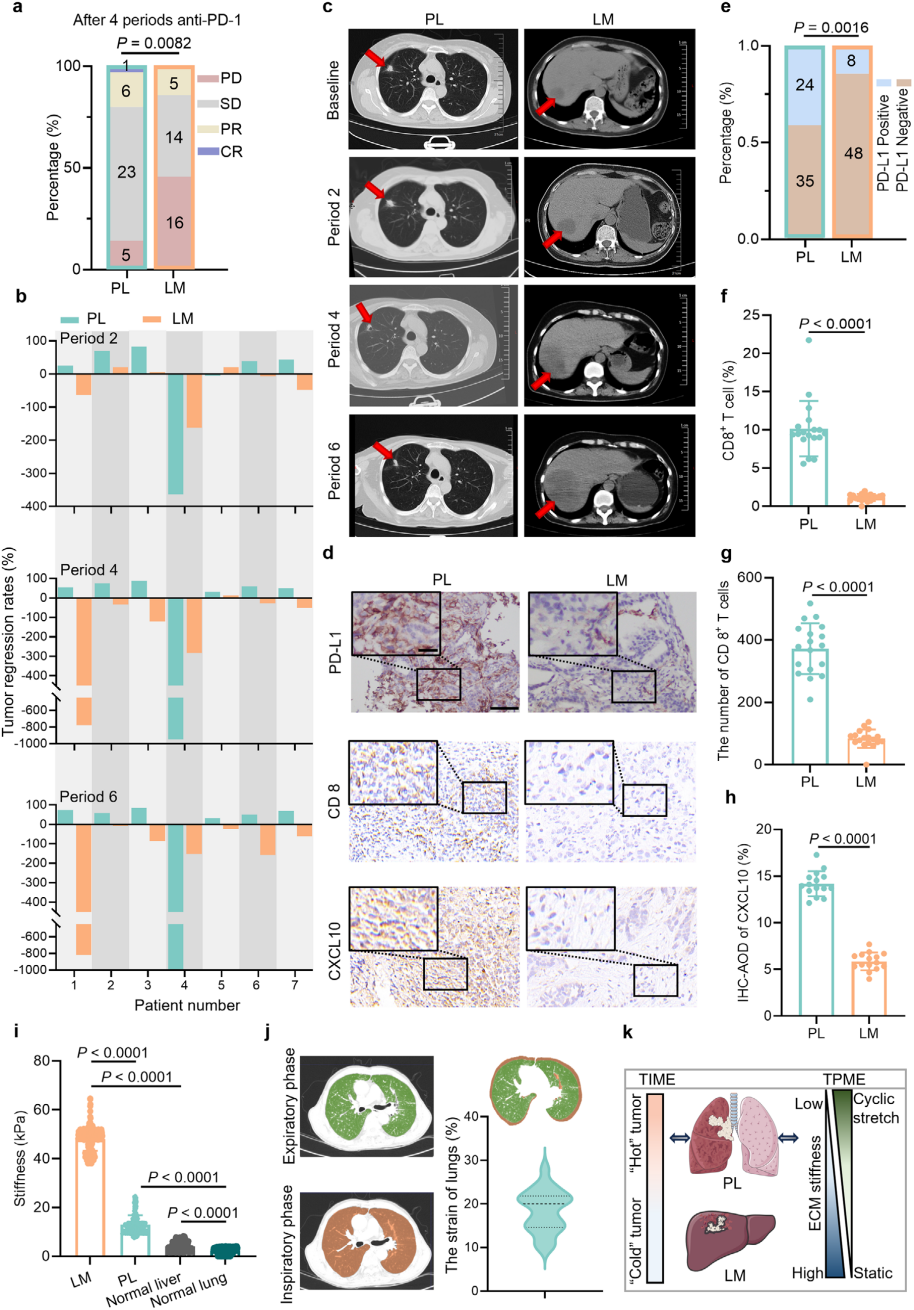
Both TIME and TPME variances correlate with poor immunotherapy response of NSCLC liver metastasis. a) Comparison of the efficacy of four periods of anti‐PD‐1 immunotherapy in PL and LM tumor sites in the same patient (n = 35). b) Comparison of tumor regression rates in PL and LM sites from the same patient at different therapy periods (n = 7). c) CT images of PL and LM sites in the same patient at baseline and in different periods of anti‐PD‐1 therapy. The red arrows indicate the location of the tumor tissue. d) The PD‐L1, CD8^+^ T cell, and CXCL10 immunohistochemical staining of PL and LM tumor tissue. The scale bars indicate 20 µm (enlarged image in the upper left corner) and 60 µm. e) PD‐L1 expression status in PL and LM sites. f) percentage, and g) absolute number of CD8^+^ T cell in PL and LM tumor tissues. h) The expression level of CXCL10 in PL and LM tumor tissues. I) The Young's modulus of patients’ tissues measured by AFM (N ≥ 7; 3 different sites for each sample, n ≥ 21). j) CT images of the inspiratory and expiratory phase, and respiratory strain range of bronchial at all levels. k) The schematic diagram of varying TIME and TPME in PL and LM sites. Data are classified as PD versus non‐PD and compared using the chi‐square test (a). Data are compared using the chi‐square test (e). Data are compared by a two‐tailed Student's *t*‐test (f‐i). In (f‐i), all data are shown as mean ± S.E.M. NSCLC, non‐small cell lung cancer; PL, primary lung cancer; LM, liver metastasis; CXCL10, chemokine C‐X‐C ligand 10; AFM, atomic force microscopy; PD, progressive disease; TIME, tumor immune microenvironment; TPME, tumor physical microenvironment.

We retrospectively analyzed clinical data from NSCLC patients with liver metastases who received at least 4 periods of anti‐PD‐1 therapy. Some patients continued beyond 4 periods according to clinical guidelines; thus evaluation data after 6 periods were also included. Details of treatment periods for individual patients are clearly indicated in **Figure** **1**b,c and summarized in Table S1 (Supporting Information). According to immune‐related response evaluation criteria in solid tumors (irRECIST), we evaluated NSCLC patients with the presence of liver metastasis treated with at least 4 periods of anti‐PD‐1 therapy at the First Affiliated Hospital of Xi'an Jiaotong University (see Table S1, Supporting Information, for the patients' demographic information). After 4 periods of treatment, liver metastatic (LM) sites show significantly worse disease progression (PD) compared to the primary lung (PL) sites (Figure 1a). Notably, patients’ response to immunotherapy was heterogeneous. For example, patient No.4 exhibited progression in both PL and LM lesions despite treatment, illustrating the innate poor responsiveness of this patient to immunotherapy (Figure 1b). Such clinical heterogeneity underscores the complexity of metastatic tumor responses to immune checkpoint inhibitors. Overall, most patients even display a substantial reduction of PL tumors, while their LM tumor grows (Figure 1b,c). The observed phenomenon is consistent with previous findings.^[^ ^26^ ^]^ Several studies have suggested that PD‐L1 expression and CD8^+^ T cell infiltration status of LM can forecast their response to anti‐PD‐1/PD‐L1 immunotherapy.^[^ ^27^ ^]^ Analysis of these markers in our histological samples revealed that the TIME of LM sites presents a “cold” tumor state, characterized by fewer PD‐L1‐positive cells (PD‐L1 expression status confirmed by two senior pathologists) (Figure 1d,e) and lower CD8^+^ T cell infiltration (Figure 1d,f,g) compared to PL sites. The TIMER (Tumor Immune Estimation Resource) database shows that CXCL10 expression is positively related to the degree of CD8^+^ T cell infiltration in NSCLC patients (Figure S1a, Supporting Information). Similarly, our histological results show that PL sites exhibit higher CXCL10 expression than LM sites (Figure 1d,h), aligning with the observed differences in CD8^+^ T cell infiltration. Such organ‐specific TIME heterogeneity is also evident in murine models (Figure S1b, Supporting Information).

Accumulating evidence shows that TPME plays a significant role in regulating TIME.^[^ ^28^ ^]^ Under normal physiological conditions, the stiffness of the liver^[^ ^29^ ^]^ is higher than the lung (2 kPa)^[^ ^30^ ^]^ due to organ‐specific heterogeneity. We therefore measured the stiffness of PL and LM tumor tissue. We found that the stiffness of LM tissue, measured from the patient's puncture tissue using atomic force microscopy (AFM), is significantly higher than PL tissue (Figure 1i). This was supported by staining results of type I collagen, where LM sites have higher deposition than PL sites (Figure S1c, Supporting Information). This observation was further corroborated in murine models, which also show increased stiffness of LM tissue (Figure S1d, Supporting Information). The lung undergoes cyclic stretch due to respiratory movements, whereas the liver remains comparatively static. Based on the statistics of all levels of bronchial respiratory strains reported in previously published studies,^[^ ^31^, ^32^ ^]^ we found that the strain range for lung tissue is approximately 18.41 ± 1.01% (Figure 1j). In summary, both TIME and organ‐specific TPME heterogeneity correlate with poor immunotherapy response of NSCLC liver metastasis (Figure 1k).

### 2.2 Cyclic Stretch Upregulates the Expression of PD‐L1 via YAP‐Mediated Piezo1 Activation

**Figure 2:**
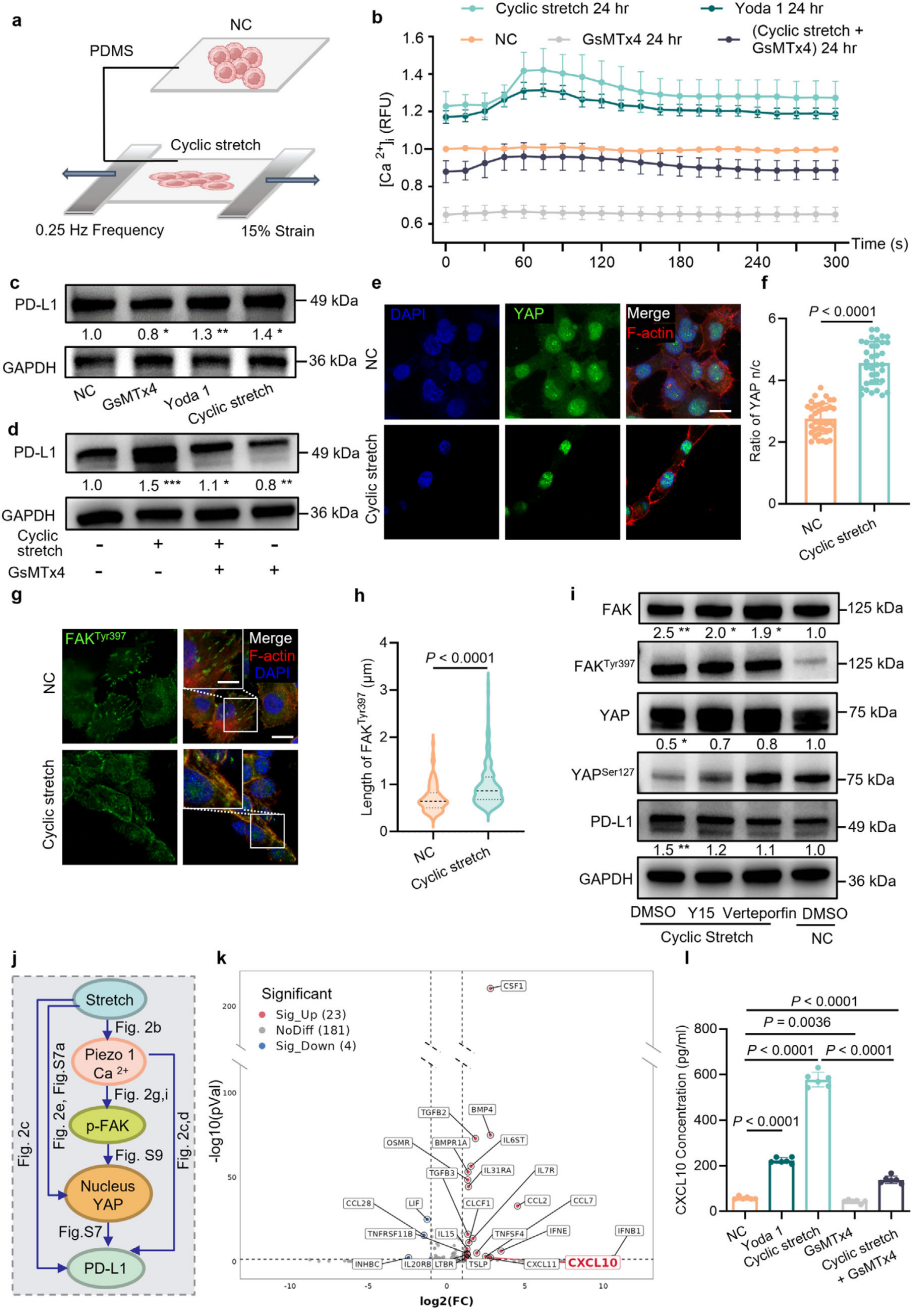
Cyclic stretch increases PD‐L1 expression via YAP‐mediated Piezo 1 activation in NSCLC cells. a) The diagram of cells cultured on static and cyclically stretched PDMS (frequency: 0.25 Hz, strain: 15%). b) Quantification of the fluorescence intensity of Ca^2+^ probes after 24‐h specific treatment on H1299 cells (N = 3). c) The western blotting of H1299 cells with certain treatments. PD‐L1 protein levels were normalized to GAPDH (N = 3). d) The western blotting of H1299 cells on static and cyclical stretch condition with/without GsMTx4 treatment. PD‐L1 protein levels were normalized to GAPDH (N = 3). The immunofluorescent images of e) YAP, and g) FAK^Tyr397^. The scale bars indicate 10 µm (images in the upper left corner) and 20 µm. Quantification of f) YAP nuclear/cytoplasmic (n/c) ratio and h) length of FAK^Tyr397^ (N ≥ 5, n ≥ 35 cells). i) The western blotting of H1299 cells cultured on static and cyclically stretched PDMS with the Verteporfin and Y15 treatment. Phosphorylation of YAP^Ser127^/FAK^Tyr397^ = gray value ratio of YAP^Ser127^/FAK^Tyr397^ over YAP/FAK. PD‐L1 protein levels were normalized to GAPDH (N = 3). j) Illustration of how cyclic stretch influences PD‐L1 expression. k) Differential gene volcano plot of environmental information processing‐related pathways. Data from RNA‐seq of H1299 cells with treatment of cyclic stretch and static condition l) The expression levels of CXCL10 in H1299 cells as determined by ELISA after treatment (N ≥ 5). Data are compared by a two‐tailed Student's *t*‐test. In (f), (h), and (i), all data are shown as mean ± S.E.M. NSCLC, non‐small cell lung cancer; PDMS, polydimethylsiloxane; RFU, relative fluorescence units; CXCL10, chemokine C‐X‐C ligand 10; ELISA, enzyme‐linked immunosorbnent assay. Yoda 1, Piezo1‐specific agonist; GsMTx4, Piezo 1 ion channel‐specific inhibitor; Verteporfin, YAP transcriptional function inhibitor; Y15, phosphorylation inhibitor of FAK^Tyr397^.

To explore whether organ‐specific TPME affects TIME, we first assessed the effect of cyclic stretch on PD‐L1 expression level in NSCLC cells. Here, we constructed an in vitro stretching device capable of modulating the amplitude and frequency of stretch strain (Figure S2a, Supporting Information). We chose 15% cyclic strain for our cell‐stretching device, guided by physiological lung strain measurements (≈18.41 ± 1.01%) in vivo. Specifically, 15% is well within the physiological range capable of inducing robust mechanotransduction without causing adverse stress‐induced cellular responses or viability loss, as established in prior mechanobiology studies.^[^ ^33^, ^34^ ^]^ We cultured H1299 cells and H1975 cells (NSCLC cell lines) on the PDMS substrate, which was stretched to mimic the respiratory strain and frequency of the lung (**Figure** **2**a). The calcium signaling pathway is significantly enriched in the KEGG enrichment analysis related to category “environmental information processing” as a result of the transcriptome sequencing of static and stretched cells (Figure S3a, Supporting Information). To validate if this enrichment correlates with stretch‐activated Piezo1 calcium channels, the change of calcium ion influx was assessed using fluorescent probes (Figure 2b; Figure S3b, Supporting Information). We observed that the 24‐h treatment of Piezo1‐specific biochemical agonist Yoda 1 and 24‐h application of cyclic stretch both led to a significant increase of calcium ion influx compared to the control group and the group with 24‐h treatment of Piezo1‐specific inhibitor GsMTx4. Meanwhile, the simultaneous treatment of GsMTx4 along with 24‐h stretching can reduce the increased calcium ion influx to a level comparable with the control group. Since there is no apparent change of Piezo1 expression (Figure S4, Supporting Information), we believe that the increase of calcium influx results from increased activation of Piezo1. Under cyclic stretch or upon Yoda 1 treatment, PD‐L1 is upregulated in both H1299 cells (Figure 2c; Figure S5, Supporting Information) and H1975 cells (Figure S6, Supporting Information). Consistently, treatment with GsMTx4 suppresses the stretch‐induced PD‐L1 upregulation in H1299 cells (Figure 2d; Figure S7, Supporting Information) and H1975 cells (Figure S8, Supporting Information). Given that Piezo1 activation facilitates nuclear translocation of YAP^[^ ^35^ ^]^ and nuclear YAP engages in the transcription of PD‐L1,^[^ ^36^ ^]^ we assessed the contribution of YAP in the regulation of PD‐L1 mediated by Piezo1. From KEGG analysis, we discovered a substantial enrichment of the hippo signaling pathway in H1299 cells after cyclic stretch (Figure S3a, Supporting Information). YAP^Ser127^ phosphorylation is a direct result of activation of the Hippo pathway, where phosphorylated YAP binds to 14‐3‐3 proteins and is sequestered in the cytoplasm, thereby reducing nuclear YAP levels. Thus, a decrease in the level of YAP^Ser127^ phosphorylation is usually accompanied by an increase in the nucleoplasmic ratio (n/c) of YAP,^[^ ^37^ ^]^ suggesting that more YAP translocates to the nucleus to exert transcriptional regulatory functions. Both the increased YAP n/c ratio and the decreased YAP^Ser127^ phosphorylation level demonstrate that cyclic stretch enhances the nuclear translocation of YAP (Figure 2e,f; Figure S9a‐b, Supporting Information) and corresponding transcriptional targets of YAP (e.g., CYR61 and CTGF) (Figure S9d, Supporting Information). Meanwhile, the inhibition of YAP transcriptional function (Verteporfin) reduces the expressions of CYR61 and CTGF, followed by the decrease of PD‐L1 expression (Figure S9e, Supporting Information). To confirm the universality of this mechanism, we conducted similar cyclic stretch experiments with another NSCLC cell line, H1975. H1975 cells displayed significantly elevated YAP n/c ratio and the expressions of CYR61 and CTGF following cyclic stretch, mediated by Piezo1‐induced YAP nuclear translocation (Figure S10, Supporting Information). This observation strongly supports the consistency and robustness of our proposed mechanotransduction mechanism in NSCLC. Since activation of Piezo1 induces phosphorylation of FAK^Tyr397^,^[^ ^38^ ^]^ we assessed the impact of cyclic stretch on FAK activity by KEGG analysis and observed the pathway enrichment of focal adhesion (FA) (Figure S9a, Supporting Information). Furthermore, the immunofluorescence image (Figure 2g,h) and western blotting (Figure 2i; Figure S8c, Supporting Information) of FAK^Tyr397^ phosphorylation confirmed the increases of FAK activity in H1299 cells under cyclic stretch. The inhibition of FAK (Y15) reduces both YAP activation and corresponding PD‐L1 expression comparable to static condition (Figure 2i; Figure S9b‐h, Supporting Information). Consistently, YAP inhibitor (Verteporfin) also suppresses the stretch‐activated upregulation of PD‐L1 (Figure 2i; Figure S9f, g, Supporting Information). Both reductions by FAK and YAP inhibition can be rescued by YAP (5SA) overexpression (Figure S10, Supporting Information). In summary, cyclic stretch increases the expression of PD‐L1 through the activation of the Piezo1‐FAK‐YAP axis.

Previous studies have shown that cyclic stretch can alter chemokine secretion.^[^ ^20^, ^39^ ^]^ In our study, we analyzed 209 genes enriched by KEGG analysis of cytokine‐cytokine receptor interaction and identified an upregulation of CXCL10 expression (Figure 2k; Figure S3a, Supporting Information). In tissue samples from NSCLC patients (Figure 1d) and murine models (Figure S1b, Supporting Information), PL sites exhibit higher CXCL10 expression than LM sites, aligning with the observed differences in CD8^+^ T cell infiltration. As reflected by ELISA results, both Yoda 1 and cyclic stretch treatments augment CXCL10 secretion in H1299 cells (Figure 2l) and H1975 cells (Figure S11b, Supporting Information), which can be reverted by Piezo1 inhibition (Figure 2l; Figure S11c, Supporting Information). The previous results indicate that activation of Piezo1 promotes YAP nuclear translocation, thus we hypothesize that higher CXCL10 secretion is associated with a higher YAP n/c ratio. This is consistent with previous findings that blocking YAP nuclear translocation significantly reduces the nuclear expression of β‐catenin as well as the mRNA levels of CTGF and CXCL10 in cholangiocytes.^[^ ^40^ ^]^ Collectively, cyclic stretch, a prominent mechanical feature of organ heterogenicity between the lung and liver, promotes PD‐L1 expression and CXCL10 secretion in NSCLC cells.

### 2.3 Optimal Matrix Stiffness Exists in the Regulation of PD‐L1

**Figure 3:**
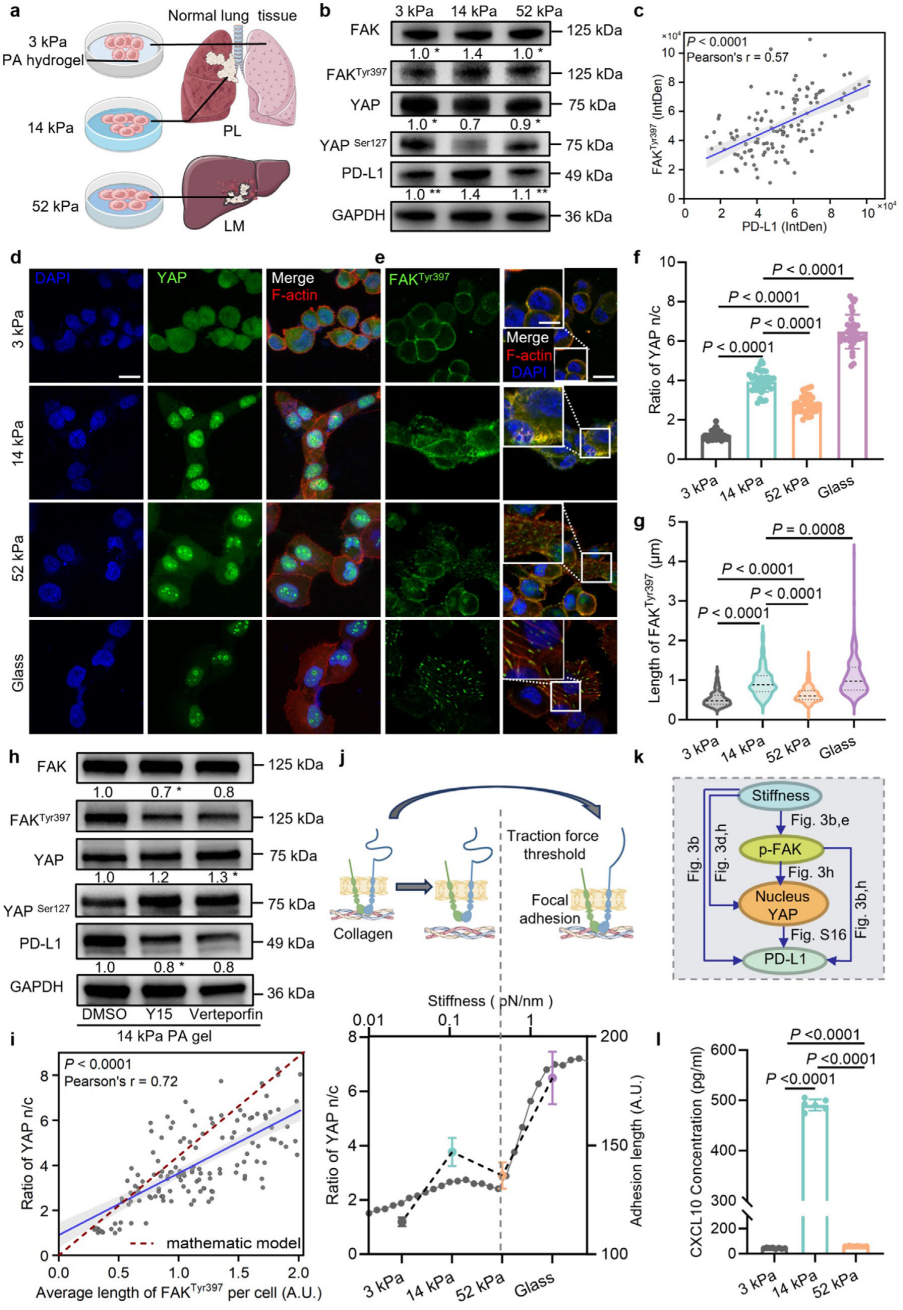
The regulation of PD‐L1 expression in NSCLC cells has an optimal matrix stiffness. a) The diagram of cells cultured on 3 kPa, 14 kPa, and 52 kPa hydrogels. b) The western blotting of H1299 cells cultured on 3 kPa, 14 kPa, and 52 kPa hydrogels. Phosphorylation of YAP^Ser127^/FAK^Tyr397^ = gray value ratio of YAP^Ser127^/FAK^Tyr397^ over YAP/FAK. PD‐L1 protein levels were normalized to GAPDH (N = 3). c) Linear analysis was performed based on FAK^Tyr397^ and PD‐L1 gray values from all western blotting results. The immunofluorescent images of d) YAP, and e) FAK^Tyr397^. The scale bars indicate 10 µm (enlarged image in the upper left corner) and 20 µm. Quantification of the f) YAP n/c ratio and g) FAK^Tyr397^ (N ≥ 5, n ≥ 35 cells). h) The western blotting of H1299 cells on 14 kPa hydrogel with Verteporfin and Y15 treatment (N = 3). i) Linear analysis of the average length of FAK^Tyr397^ per cell and YAP n/c ratio (blue line). The scaling function is constructed by a mathematical model (red dotted line). j) The top diagram shows the molecular clutch model of force transmission, and the bottom one shows the simulation results of the mathematical model and evolution of the YAP n/c ratio along with stiffness change. k) Illustration of how ECM stiffness influences PD‐L1 expression. l) The expression levels of CXCL10 in H1299 cells (N ≥ 5). Data are compared by a two‐tailed Student's *t*‐test. In (f), (g), and (l), all data are shown as mean ± S.E.M. NSCLC, non‐small cell lung cancer; CXCL10, chemokine C‐X‐C ligand 10; Verteporfin, YAP transcriptional function inhibitor; Y15, phosphorylation inhibitor of FAK^Tyr397^.

Next, we explored the impact of ECM stiffness on PD‐L1 expression of NSCLC cells. Here, we constructed three different stiffness hydrogels, based on our prior measurements (Figure 1i), mimicking native tissue stiffness of normal lung (3 kPa), primary lung cancer (14 kPa), and lung cancer liver metastasis (52 kPa) (**Figure** **3**a). Previous studies have suggested that PD‐L1 expression in NSCLC cells is enhanced by increasing stiffness.^[^ ^19^ ^]^ However, our findings demonstrate that both H1299 cells (Figure 3b; Figure S12a, d, Supporting Information) and H1975 cells (Figure S13a,b,d, Supporting Information) exhibit the highest PD‐L1 expression on 14 kPa hydrogel. This suggests the existence of an optimal matrix stiffness for PD‐L1 expression in both cell lines (Figure S14, Supporting Information). Given that p‐FAK‐dependent nuclear translocation of YAP positively correlates with PD‐L1 expression, we assessed the expressions of YAP activity and the level of FAK^Tyr397^ phosphorylation and found a peak in H1299 (Figure 3b; Figure S12b,c,e, Supporting Information) and H1975 cells (Figure S13a,c, Supporting Information) cultured on 14 kPa hydrogel. A positive correlation is shown in a linear analysis of the relative integrated density of PD‐L1 and FAK^Tyr397^ from all western blotting results (Figure 3c).

KEGG enrichment analysis from subsequent transcriptome sequencing of cells cultured on three stiffness conditions points toward enrichment of the Hippo signaling pathway (Figure S15a, Supporting Information). Taking the cells cultured on glass slides as the control group with extremely high stiffness, we measured the YAP nuclear localization and the length of FAK^Tyr397^ in H1299 cells across four stiffness levels by immunofluorescence staining (Figure 3d‐e). Intriguingly, we observed that the YAP n/c ratio and the phosphorylation level of FAK^Tyr397^ approach a plateau at 14 kPa, then increase again following a drop at 52 kPa (Figure 3f‐g). On the optimal 14 kPa hydrogel, the inhibition of FAK reduces both YAP activation and corresponding PD‐L1 expression (Figure 3h; Figure S16a,b, Supporting Information). Similarly, YAP inhibition also suppresses PD‐L1 expression (Figure 3h; Figure S16a,c, Supporting Information). Overexpression of YAP (5SA) recovers the reduction of PD‐L1 level induced by FAK inhibition (Figure S16d–g, Supporting Information).

To elucidate this observation, we employed mathematical models for simulation and validation. Using the length of FAK^Tyr397^ to represent the FA length as described previously,^[^ ^41^ ^]^ we performed a linear regression analysis of FA length with the YAP n/c ratio and found a direct correlation between FA length and YAP (Figure 3i). Based on this scaling function, we developed a modified motor‐clutch model (see the mathematical model part in method for the details of the model). The simulation results show that the YAP n/c ratio increases with increasing stiffness below optimum 14 kPa, while it drops mildly before a dramatic increase over 14 kPa (Figure 3j). We hypothesize that talin, a protein component of FA, may play a role in this association. Hence, we used the motor‐clutch model to explain this optimal matrix stiffness for mechanotransduction. By simulating the molecular dynamics at the integrin‐matrix interface, our motor‐clutch model shows that (*i*) at low matrix stiffness (<14 kPa), the on‐rate of integrin clutch gently increases with increasing ECM stiffness, leading to the increases of adhesion length and YAP nuclear translocation; (*ii*) at intermediate stiffness (14 kPa–52 kPa), integrin clutch trends to unbind with matrix due to a large bond tension and loading rate, thus leading to the decreases of adhesion length and YAP nuclear translocation; (*iii*) finally, at high stiffness (>52 kPa) with a larger loading rate, due to the clutch reinforcement of talin‐vinculin complex, the on‐rate of integrin clutch increases again (due to the average force per monomer to drop), leading to the increases of adhesion length and YAP nuclear translocation (Figure 3j).

Based on the preceding research outcomes, our investigation persisted in examining the impact of varying stiffness on chemokines. Through an analysis of the transcriptome sequencing outcomes, we observed an enrichment of the cytokine‐cytokine receptor interaction (Figure S15a, Supporting Information). We thus analyzed the chemokines produced by cells cultured on varied ECM stiffness. As expected, both H1299 cells (Figure 3l) and H1975 cells (Figure S15b, Supporting Information) cultured on the 52 kPa hydrogel secrete less CXCL10 than those on the 14 kPa hydrogel, consistent with our earlier observation of reduced CXCL10 secretion in LM sites (Figure 1d). All these results suggest the existence of optimum stiffness in regulating PD‐L1 expression within the physiologically relevant range.

### 2.4 Piezo1 Activation Dominantly Upregulates the Expression of PD‐L1 in Heterogeneous TPME of NSCLC Liver Metastasis

**Figure 4:**
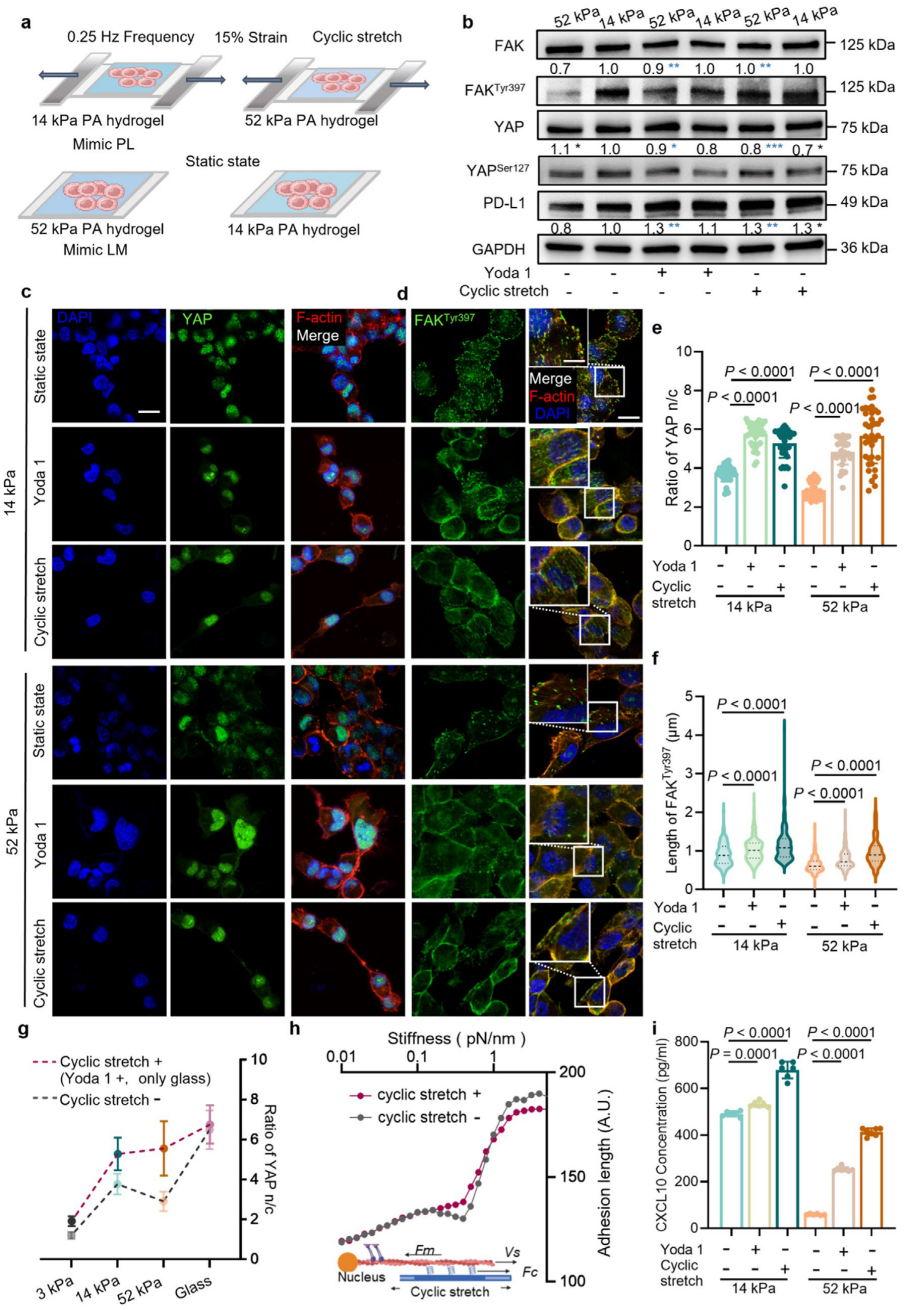
Piezo1 activation dominates TPME‐related upregulation of PD‐L1 in NSCLC liver metastasis. a) The diagram of cells cultured on the stretching platform with tunable matrix stiffness. b) The western blotting of H1299 cells cultured on 14 kPa and 52 kPa hydrogels treated with Yoda 1 or cyclic stretch. *, The comparison between the control group and groups treated with Yoda 1 or cyclic stretch on 14 kPa (black) and 52 kPa (dark blue). Phosphorylation of YAP^Ser127^/FAK^Tyr397^ = gray value ratio of YAP^Ser127^/FAK^Tyr397^ over YAP/FAK. PD‐L1 protein levels were normalized to GAPDH (N = 3). The immunofluorescent images of c) YAP, and d) FAK^Tyr397^. The scale bars indicate 10 µm (enlarged image in the upper left corner) and 20 µm. e) Quantification of the YAP n/c ratio (N ≥ 5, n ≥ 35 cells). f) Quantification of the length of FAK^Tyr397^ in H1299 cells (N ≥ 5, n ≥ 35 cells). g) The YAP n/c ratio alteration of cells cultured on various stiffness hydrogels with and without cyclic stretch (cells on glass slides were treated with Yoda 1). h) Schematic illustration of mathematical model and prediction of adhesion length change in cells cultured on various stiffness matrices coupled or uncoupled with cyclic stretch. i) The expression levels of CXCL10 in the supernatant of H1299 cells (N ≥ 5). Data are compared by a two‐tailed Student's *t*‐test. In (b), (e), (f), and (i), all data are shown as mean ± S.E.M. NSCLC, non‐small cell lung cancer; Yoda 1, Piezo1‐specific agonist; CXCL10, chemokine C‐X‐C ligand 10.

In order to assess the impact of metastasis‐induced TPME heterogenicity on TIME, we further evaluated the two key TPME changes in NSCLC liver metastasis, namely the combination of matrix stiffness with cyclic stretch. Through orthogonal experiments of stiffness‐combined cyclic stretch (**Figure** **4**a; Figure S2b, Supporting Information), we observed that Piezo1 activation (either by Yoda 1 or cyclic stretch) increased the PD‐L1 expression in H1299 cells (Figure 4b; Figures S17a,b and S18, Supporting Information) and H1975 cells (Figure S19, Supporting Information) cultured on both 14 kPa and 52 kPa hydrogels. The alteration in expression can be attributed to the nuclear localization of YAP and the activation of FAK (Figure 4b‐d; Figure S17c‐e, Supporting Information), as evidenced by our earlier findings (Figures 2e‐i and 3d‐h). Consistent with our in vitro mechanotransduction model, immunohistochemistry in mouse tumors confirmed significantly increased levels of phosphorylation of FAK^Tyr397^ and nuclear YAP in PL compared to LM, reinforcing our proposed mechanistic connection between mechanical cues and tumor immune microenvironment changes (Figure S22, Supporting Information). Remarkably, with the activation of Piezo1, we observed that the phenomenon of the optimal stiffness governing PD‐L1 expression disappeared, and PD‐L1 expression exhibited a nearly linear increase across various matrix stiffnesses. Changes in the YAP n/c ratio and FAK^Tyr397^ phosphorylation level are also consistent with this tendency (Figure 4e,f). We think that this shift (Figure 4g) originates from the unfolding of integrins as induced by cyclic stretch, which subsequently amplifies the average force, masking the concavity. After incorporating this modification into our mathematical model, the updated adhesion length‐stiffness curve aligns well with our experimental observations (Figure 4h).

Furthermore, we checked if CXCL10 secretion showed a similar change after activating Piezo1 by cyclic stretch or Yoda 1 treatment. We found a continuous and evident increase in CXCL10 across cells cultured on matrix of varied stiffness (Figure 4i), with the loss of optimal matrix stiffness (Figure 3l). Taken together, the PD‐L1 expression and CXCL10 secretion are dominantly regulated by Piezo1 activation in organ‐specific TPME heterogenicity of NSCLC liver metastasis.

### 2.5 Activating Piezo1 Could Serve as a Promising Strategy for Improving Immunotherapy Efficacy in Liver Metastasis

**Figure 5:**
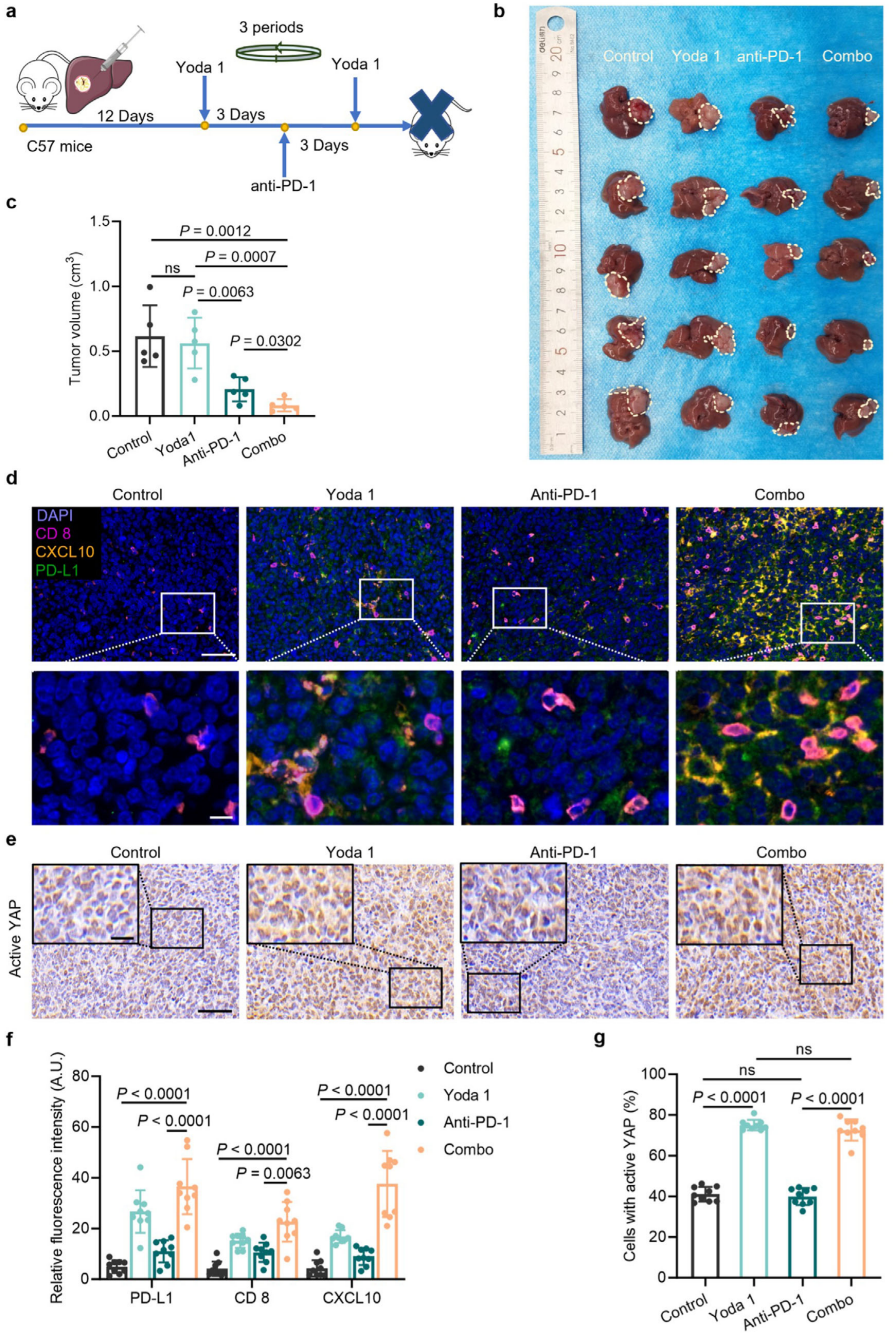
Piezo1 activation could be a promising strategy for improving immunotherapy efficacy in lung cancer liver metastasis. a) Diagram depicts the construction of the murine model with schedules of drug treatment. b) Image of liver metastatic tumors. c) Quantification of the tumor volume (N = 5). d) Multiplex immunohistochemistry. The scale bars indicate 20 µm (enlarged image) and 60 µm. e) The active YAP immunohistochemistry staining. The scales indicate 20 µm (enlarged image in the upper left corner) and 60 µm. f) Quantification of the fluorescence intensity of each marker (each treatment group, N = 3; 3 different sites for each sample, n = 9). g) Quantification of the cells with active YAP (each treatment group, N = 3; 3 different sites for each sample, n = 9). Data are compared by a two‐tailed Student's *t*‐test. In (c), (f), and (g) data are shown as mean ± S.E.M. Yoda 1, Piezo1‐specific agonist.

Since Piezo1 activation increases the PD‐L1 expression and CXCL10 secretion in H1299 cells cultured on a 52 kPa hydrogel (mimicking the TPME of liver metastasis), we hypothesize that Piezo1 activation can fortify the potency of anti‐PD‐1 therapy at the site of liver metastasis. Employing the modeling approach of the colon cancer liver metastasis murine model,^[^ ^6^ ^]^ we administered LLC cells (Lewis lung carcinoma line) beneath the liver capsule of C57/BL mice and subsequently divided the mice into four groups for different treatments (control, Yoda 1 treatment group, anti‐PD‐1 treatment group, combo treatment group) (**Figure** **5**a). Following three treatment cycles, we euthanized the mice and excised their livers for tumor growth evaluation (Figure 5b), and observed negligible therapeutic difference between the control and the Yoda 1 treatment group (Figure 5c). The dosage of Yoda 1 has been proven to be noncytotoxic in vitro (Figure S20, Supporting Information). In contrast, when comparing the anti‐PD‐1 treatment group with the combined treatment group (anti‐PD‐1 + Yoda 1), we observed a significant reduction (40.38%) in the volume of liver metastasis tumor (206.50 ± 41.71 versus 83.38 ± 21.22 mm^3^) (Figure 5c).

To assess whether the improved therapeutic outcome is attributed to changes in TIME, we performed multi‐color immunofluorescence staining on tissue samples of murine LM sites. In comparison to the control group, the Yoda 1 treatment group has higher levels of PD‐L1 expression, CXCL10 secretion, and CD8^+^ T cell infiltration (Figure 5d,f). The anti‐PD‐1 monotherapy group exhibits increased CD8^+^ T cell infiltration than the control, but its efficacy decreases in comparison to the combined treatment group (Figure 5d,f). When compared to the combined treatment group, both CXCL10 expression and CD8^+^ T cell infiltration are lower in the Yoda 1 treatment group, due to the less death of tumor cells after Yoda 1 treatment. As a result, the reduced release of tumor antigens fails to effectively promote CD8^+^ T cell infiltration (Figure 5d,f). Despite increased CD8^+^ T cell infiltration, the anti‐PD‐1 therapy group is less effective than the combined treatment group, mainly due to the lack of PD‐L1 expression in TIME. Previous studies have established that TIME featuring CD8^+^ T cell infiltration without PD‐L1 expression exhibits reduced sensitivity to anti‐PD‐1/PD‐L1 immunotherapy.^[^ ^42^ ^]^ In the combination therapy group, the release of large amounts of tumor antigens from tumor cells killed by anti‐PD‐1 therapy, as well as the increased secretion of CXCL10 induced by Yoda 1, will recruit more CD8^+^ T cells and release more tumor antigens, thus further achieving a cascade response in the anti‐tumor immune cycle. Hence, the combination therapy leads to a “hot” tumor status in TIME (Figure 5d,f). To verify whether the observed transformation in TIME originates from the previously identified mechanism, i.e., Piezo1 activation enhanced YAP nuclear translocation, we compared the active YAP expression across the treatment groups. Consistently, the percentage of nuclear YAP localization is significantly higher in the two groups with Yoda 1 treatment (Figure 5e,g). Taken together, the activation of Piezo1 promotes PD‐L1 expression and CD8^+^ T cell infiltration in the liver metastatic TIME, converting it to a “hot” tumor that responds better to anti‐PD‐1/PD‐L1 immunotherapy. These findings show the potential of targeting Piezo1 as a novel treatment method for synergistically improving immunotherapy efficacy.

## 3 Discussion

Our study primarily aims to elucidate the diminished response of NSCLC liver metastasis to anti‐PD‐1/PD‐L1 immunotherapy from the aspect of organ‐specific heterogeneity. We discovered that discrepancies of TPME in LM sites, characterized by stiffened ECM and loss of cyclic stretch (Figure 1i,j; Figure S1c, d, Supporting Information), profoundly affect the TIME (Figure 1d‐h; Figure S1b, Supporting Information). Here, we identified the crucial role of Piezo1 in TPME‐dependent mechanobiological regulation of TIME in NSCLC. Notably, both mechanical and biochemical activations of Piezo1 upregulate the expression of PD‐L1 and secretion of CXCL10 in NSCLC cells through YAP activation mediated by p‐FAK (Figures 2c‐i,l and 4b‐f,i), and hence improve immunotherapy response. In combination with anti‐PD‐1 antibody, the Piezo1 agonist successfully converts the TIME of LM sites from a “cold” status to a “hot” status in a murine model and significantly improves immunotherapy efficacy (Figure 5b,d).

Cyclic stretch stands out as a key mechanical difference in NSCLC liver metastasis. Our study shows that cyclic stretch increases the expression of PD‐L1 (Figure 2c; Figure S5a, c, Supporting Information) and secretion of CXCL10 (Figure 2l; Figure S11b, Supporting Information), fostering a “hot” TIME in primary tumors with improved immunotherapy response, as shown in the clinical analysis of primary and liver metastasis NSCLC patients (Figure 1b). Moreover, cyclic stretch affects immune dynamics and cell activity through mechanotransduction in disease and cancer.^[^ ^43^ ^]^ Such mechanical cue can trigger the release of the NLRP3 inflammasome, a lung cancer promoting protein complex,^[^ ^44^ ^]^ by epithelial cells after lung injury,^[^ ^45^ ^]^ which facilitates TGF‐β1 secretion from mast cells in the development of lung fibrosis.^[^ ^46^ ^]^

In addition, we also explore the role of ECM stiffness, another TPME difference, in NSCLC liver metastasis. Our work reveals the existence of an optimal stiffness threshold that governs the expression of PD‐L1 and secretion of CXCL10 (Figure 3b,l), consistent with the YAP n/c ratio (Figure 3d,f). The phosphorylation of FAK increases cell contraction, sequentially raises nuclear tensile stress, and deforms the nuclear pore complex (NPC), eventually resulting in more entry of YAP into the nucleus. This hypothesis is supported by our observation (Figure S9I, Supporting Information) that longer phosphorylated FAK is accompanied by larger nuclear deformation, which is consistent with the findings of other studies.^[^ ^47^, ^48^, ^49^ ^]^ Thus, we assess the size and activation of FAK (Figure 3e,g) and find a positive relationship between p‐FAK‐mediated YAP nuclear translocation and PD‐L1 expression (Figure 3c,i). We propose that this optimal matrix stiffness “14 kPa” originates from unique mechanosensory characteristics of integrin with matrix stiffness, as earlier studies suggested (56). Regarding the progression and treatment of lung cancer, this interesting observation explains the collapse of immunotherapy once liver metastasis occurs. The increased ECM stiffness reprograms the immunophenotype of NSCLC cells, resulting in “cold” TIME and corresponding poor immunotherapy response. Employing a self‐build stretch platform with adjustable matrix stiffness, we also disclose that the optimal matrix stiffness for PD‐L1 and CXCL10 regulation vanishes under cyclic stretch (Figure 4b,i). We speculate that cyclic stretch constantly activates FAK in a linear pattern, resulting in a monotonic increase of p‐FAK, nuclear YAP, and consequently PD‐L1. When integrated with matrix stiffness, cyclic stretch overrides the multimodal elevation regulated by matrix stiffness with the consistent increment (Figure 4b,e,f), which is theoretically confirmed by our mathematical model (Figure 4h). Regarding the mechanosensitive protein responsive for activating FAK, talin, an adhesion‐enriched cytoskeletal protein, emerges as a prime candidate. This protein undergoes structural changes upon experiencing forces exceeding the 5 pN threshold,^[^ ^50^ ^]^ constantly maintaining cellular contractility. This phenomenon implies that cyclic stretch is crucial in altering the expression of PD‐L1 and secretion of CXCL10. While comparing their expressions with separate blockade of Piezo1 and FAK activity may offer additional insights, it is still hard to definitively clarify the individual contributions of cyclic stretch and matrix stiffness since the mechanosensory proteins interact with each other. Although FAK^Tyr397^ phosphorylation was significantly inhibited by Y15 treatment under cyclic stretch conditions (Figure 2i), complete inhibition was not observed, likely due to alternative mechanosensitive signaling pathways activated by strong mechanical stimuli.^[^ ^51^ ^]^ Additionally, our data indicate a positively nonlinear relationship between FAK activation and downstream YAP nuclear localization and PD‐L1 expression (Figure 4b). This nonlinear relationship has been explicitly quantified and illustrated (Figure S19e, Supporting Information), reflecting the complexity and redundancy inherent in mechanosensitive signaling pathways. Stretch mechano‐sensors, notably Piezo1,^[^ ^15^ ^]^ get activated by cellular tensile stress prompted by ECM stiffness.^[^ ^52^ ^]^ Similarly, stretch promotes the formation, maturation, and activity of FAs.^[^ ^51^ ^]^ Recent studies also showcase that Piezo1 not only physically links with the cytoskeleton through the cadherin‐β‐catenin complex^[^ ^53^ ^]^ but also orchestrates FAK signaling in a dual‐mode fashion,^[^ ^54^ ^]^ further entangling this intricate web of regulation.

**Figure 6:**
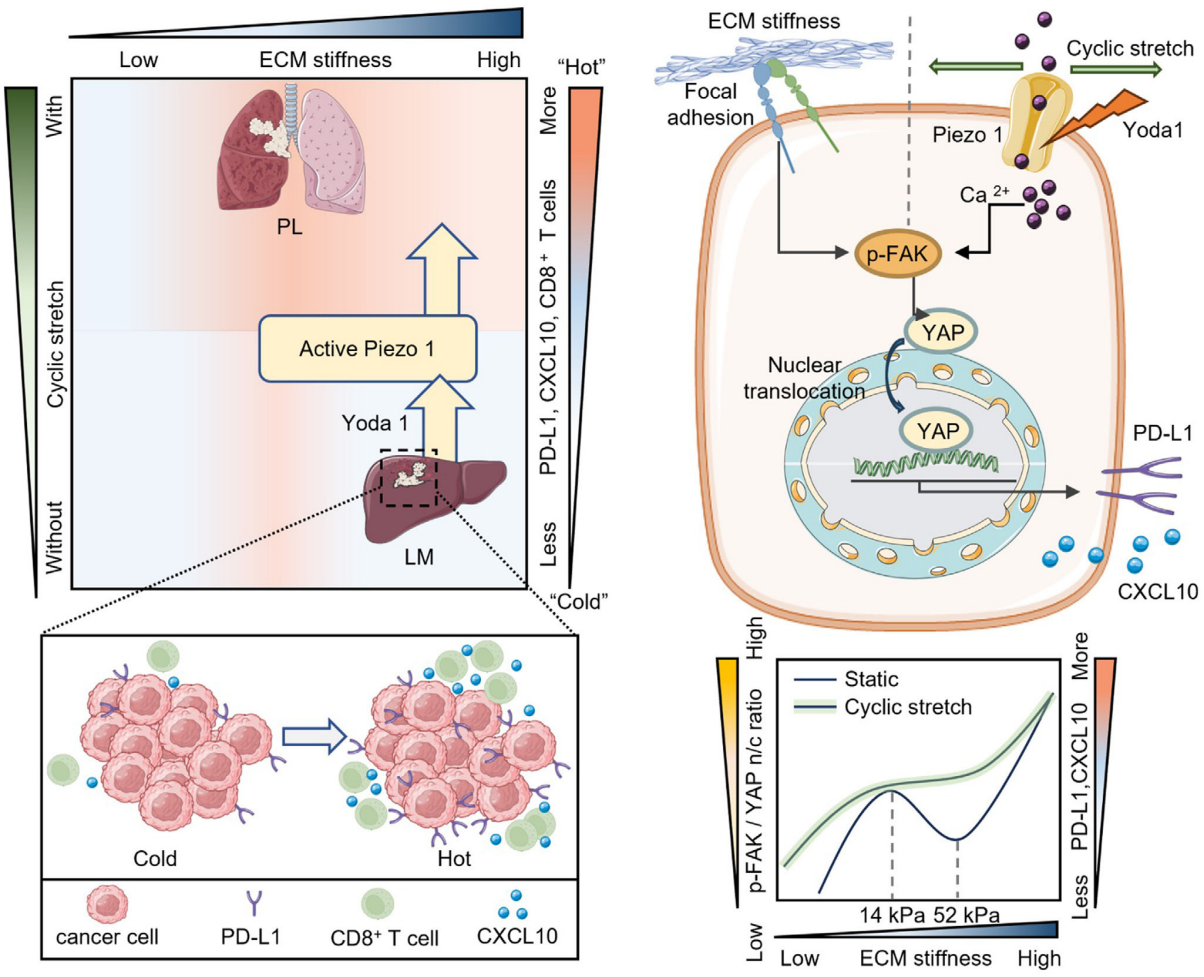
The graphic abstract of this study. PL, primary lung cancer; LM, liver metastasis; CXCL10, chemokine C‐X‐C ligand 10; ECM, extracellular matrix; Yoda 1, Piezo1‐specific agonist.

Here, we present the first evidence highlighting the critical role of using Piezo1 as a mechano‐chemical coupled target in reverting NSCLC LM sites from “cold” to “hot” TIME (**Figure** **6**). Interestingly, YAP plays an essential role in this anti‐tumor regulation of Piezo1, contrary to its known role in promoting cancer progression. Actually, an increasing amount of evidence has shown the suppressive role of YAP in cancer progression, such as the low expression and activity of YAP in various types of cancer including lung cancer,^[^ ^55^ ^]^ and forced activation of YAP is shown to reduce cell growth or trigger cell apoptosis.^[^ ^55^, ^56^ ^]^ Meanwhile, similar to our findings, it has been reported that YAP/TAZ‐activated liver cancer cells can induce antitumor immune responses.^[^ ^57^ ^]^ As indicated by the improved immunotherapy efficacy by the synergistic deployment of Piezo1 agonist with anti‐PD‐1 antibodies, activation of Piezo1 can be employed as an approach to target TPME to boost the immunotherapy efficacy of NSCLC liver metastasis. However, before extrapolating Piezo1 activation to a broader spectrum of cancers, solid experimental and clinical evaluations are essential, especially considering cancers with different cyclic stretch profiles, like lung cancer bone metastasis, colon cancer lung metastasis, and liver cancer lung metastasis. Moreover, the prognostic implications of Piezo1 expression and activation are cancer‐specific. For example, elevated Piezo1 expression has been linked to decreased patient survival in multiple cancer types (e.g., colon cancer and glioma).^[^ ^38^, ^58^, ^59^ ^]^ Conversely, NSCLC patients manifesting higher Piezo1 expression are shown to own better survival, and loss of Piezo1 is proven to accelerate NSCLC progression and NSCLC cell migration.^[^ ^60^ ^]^ Hence, the multifaceted character of Piezo1 cannot be overlooked in its clinical translation although Piezo1 is a negative regulator of NSCLC progression.

In this study, we observe that Piezo1 activation could upregulate the expression of PD‐L1 in heterogenous TPME of NSCLC liver metastasis. However, the upregulation of PD‐L1 expression may lead to the immune escape of tumor cells, which represents a significant challenge in clinical practice. PD‐L1 characterization in tumor tissue is a necessary clinical assessment for anti‐PD‐1 or anti‐PD‐L1 therapy, regardless of the chosen treatment.^[^ ^61^ ^]^ Higher percentages of PD‐L1 positive tumor cells (over 50%) and PD‐L1 expression are associated with better clinical outcomes in NSCLC patients receiving anti‐PD‐1 therapy.^[^ ^62^, ^63^, ^64^, ^65^ ^]^ We realize that the higher PD‐L1 level assists tumor cells evade immune surveillance during cancer development.^[^ ^66^ ^]^ However, we believe that upregulation of PD‐L1 is not the sole contributor to improved immunotherapy efficacy induced by Piezo1 agonist in our study, since upregulating PD‐L1 is shown to switch “cold” tumors to “hot” tumors and improve the efficacy of anti‐PD‐1/PD‐L1 immunotherapy.^[^ ^67^, ^68^ ^]^ CD8^+^ T cell infiltration is also a key factor impacting immunotherapy efficacy. The tumor tissues with both higher levels of PD‐L1 and more CD8^+^ T cell infiltration appear better immunotherapy response than that only with either higher PD‐L1 level or more CD8^+^ T infiltration.^[^ ^69^ ^]^ In addition, simultaneous enhancement of the PD‐L1 expression and infiltration of CD8^+^ T cell has been applied to improve the immunotherapy response of tumor tissues,^[^ ^70^, ^71^ ^]^ which is consistent with our observation (Figure 5b‐d). Taken together, in our study, we not only consider increasing PD‐L1 expression in NSCLC cells at LM sites, but also try to promote CD8^+^ T cell infiltration in the microenvironment. Therefore, we focus on the chemokine CXCL10, which recruits CD8^+^ T cells,^[^ ^72^ ^]^ and discover that NSCLC cells secrete more CXCL10 after Piezo1 activation. Our in vitro results confirm that upregulation of PD‐L1 expression in liver metastasis along with increased CD8^+^ T cell infiltration has an ameliorating effect on anti‐PD‐1 immunotherapy. Beyond CD8^+^ T cells, various other immune populations, including regulatory T cells (T_regs_), myeloid‐derived suppressor cells (MDSCs), and tumor‐associated macrophages (TAMs), significantly shape the tumor immune microenvironment and influence immunotherapy efficacy.^[^ ^73^, ^74^ ^]^ Future investigations should explore how mechanical cues such as cyclic stretch and ECM stiffness specifically modulate these additional cell subsets, further clarifying the comprehensive mechanotransductive landscape influencing tumor immunity. Our choice of human‐derived NSCLC cell lines (H1299, H1975) for in vitro mechanistic studies and murine LLC cells for in vivo experiments was deliberate. LLC cells, originating from syngeneic mouse tumors, offer significant advantages in studying tumor‐immune interactions due to immune compatibility with host mice, thus effectively modeling complex tumor‐host immune dynamics.^[^ ^75^, ^76^ ^]^ Nevertheless, we acknowledge interspecies differences may exist, and future validation using patient‐derived xenografts would further enhance clinical relevance.

Our study, due to its relatively limited patient sample size, did not differentiate between squamous and non‐squamous NSCLC histology in terms of TPME and TIME differences. Recent literature has highlighted significant distinctions in tumor microenvironment characteristics and immunotherapy responsiveness between these pathological subtypes.^[^ ^77^ ^]^ Larger cohort studies specifically examining these subtypes would be beneficial to fully understand TPME and TIME distinctions in various histology subtypes of NSCLC. Our work emphasizes the profound influences of organ‐specific TPME heterogeneity on different immunotherapy outcomes between PL and LM sites (Figure 6). It is pivotal to acknowledge that the immunotherapy efficacy depends on many factors, including the heterogeneous composition of immune and stromal cells across metastatic sites,^[^ ^8^, ^78^ ^]^ alterations in cellular functionalities within these sites,^[^ ^79^ ^]^ metabolic disparities spanning distinct metastatic sites,^[^ ^80^ ^]^ and the challenges associated with the delivery of immunotherapy agents. In addition to TPME, we must consider these factors, to understand the different immune responses of cancer cells in primary versus metastatic sites. Our work exposes only the tip of the iceberg of the organ heterogeneity between PL and LM sites. Thus, multi‐omics analysis methods, which have developed rapidly in recent years, are powerful tools to further explore the organ heterogeneity of different metastatic sites.^[^ ^81^ ^]^ The essential idea of our work‐improving immunotherapy through modulation of TPME‐coincides with the novel concept of mechanomedicine, which diagnoses, prevents, and cures disease through biomechanical and mechanobiological approaches. Several recent studies have proven the efficacy of interfering strategies enlightened by mechanomedicine in the treatment of cancers,^[^ ^82^ ^]^ refractory skin diseases,^[^ ^83^ ^]^ and cardiovascular diseases,^[^ ^84^ ^]^ among others.

## 4 Conclusion

In summary, our study underscores the crucial role of organ‐specific TPME heterogeneity in influencing immunotherapy outcomes, especially between primary lung sites and liver metastatic counterparts. Furthermore, our study indicates the stretching‐induced elevation of Piezo1 activity can modulate TIME via YAP activation to re‐sensitize poor‐responsive NSCLC cells to immunotherapy. This work implies the potential clinical application of Piezo1 agonist in combination with immunotherapy and makes a way to understand how TPME mechano‐biologically regulates TIME (Figure 6).

## 5 Experimental Section

### Clinical Information and Tissue Collection

Patient samples were sourced from the Department of Medical Oncology, First Affiliated Hospital of Xi'an Jiaotong University, China. Ethical approval was granted by Xi'an Jiaotong University (No. 2022‐1657), and all patients provided informed consent.

### AFM Measurement

All atomic force microscopy (AFM) indentations were performed with a 10‐µm diameter polystyrene bead affixed to a cantilever with spring constant of 0.35 N m^−1^. The AFM force maps typically 100 × 100 µm with 10 × 10 pixels, were acquired using contact mode force mapping. The Poisson's ratio of 0.5 was applied to calculate the Young's modulus.

### PDMS Preparation and Cell Culture Hydrogel Preparation

The stretchable membranes were prepared with PDMS base and crosslinker at a 10:1 ratio. The PDMS membranes were coated collagen overnight before seeding cells. Stretching experiments were carried out 24 hr post‐seeding for 24 hr. PAA hydrogels were prepared as described.^[^ ^85^ ^]^ The stiffness of PAA hydrogels was characterized with Bose ElectroForce 3200 instrument.

The construction method of PAA hydrogels substrate on PDMS membrane was improved according to the literature.^[^ ^86^, ^87^ ^]^ The PDMS substrates were incubated in a benzophenone solution in acetone (10 wt %) for 15 min under a nitrogen atmosphere and dried. And the coated PDMS substrates were washed in ethanol for 30 min. The polyacrylamide (PAA) hydrogel monomer solutions were prepared as described previously. The monomer solution was then loaded into the PDMS stretchable membranes and then exposed to ultraviolet light for 10 min. After the polymerization, the hydrogels coated PDMS substrates were washed in ultrapure water three times. And then the hydrogels were disposed as described previously.

### Calcium Imaging

H1299 cells were incubated with Fluo‐4 AM following the manufacturer's protocols after 24‐h treatment (cyclic stretch, Yoda 1, GsMTx4, etc.). The residual Fluo‐4 AM was removed by washing with HBSS buffer. Fluorescent imaging was performed by confocal scanning microscope with 40× objective lens. Images were captured every 15 s for 6 min. After the first image was captured, HBSS buffer was added dropwise to provide Ca^2+^ for Fluo‐4 AM imaging.

### Western Blotting

Total protein was extracted from tissues and cells by radio immunoprecipitation assay lysis buffer. After concentration determination by BCA Protein Assay Kit, the protein was mixed with loading buffer and heated to 100 °C for 10 min for denaturation. Protein with equal mass was loaded into polyacrylamide gels and separated by electrophoresis. Protein was then transferred to polyvinylidene difluoride membranes. The membranes were blocked in blocking buffer and incubated with primary antibodies diluted in Universal Antibody Dilution Buffer overnight at 4 °C, containing the first antibodies. After washing by TBS with 1% Tween, the membranes were incubated with HRP‐labeled goat anti‐mouse/rabbit secondary antibodies for 30 min at room temperature. The membranes were washed for three times with TBST before chemiluminescence. The blots were developed via the Light Chemiluminescence Kit. The antibodies and reagents used are shown in Tables S8 and S6 (Supporting Information). All blots were conducted with 3 biological replicates and analyzed with software Image J.

### Animal Experiments

For lung tumor murine models, LLC cells (2 × 10^6^) were tail‐vein injected in six‐week‐old C57BL/6 mice. For liver metastasis murine models, LLC cells (2.5 × 10^6^) were intrahepatically injected as described previously.^[^ ^6^ ^]^ After 12 days, mice were randomly divided into four groups. The animal treatment protocol and schedule are shown in Table S2 (Supporting Information). On day 30, all mice were euthanized and their livers were excised for evaluation. The ethical approval of animal studies was obtained from the Ethics Committee of the Health Science Center of Xi'an Jiaotong University (No. 2022‐1657).

### Mathematical Model

The detailed description of modified motor‐clutch model is included in supplementary information.

### Statistical Analyses

All data are shown as mean ± S.E.M. If data processing required normalization, it has been described in the figure legends. The number of experimental repetitions (N) and the sample size for each statistical analysis (n) are described in the figure legends. Statistical analyses were performed using two‐tailed Student's *t*‐test. *P* < 0.05 was considered a significant difference. The software used for statistical analysis is Graphpad Prism 10.2.3.

## Conflict of Interest

The authors declare no conflict of interest.

## Author Contributions

T.Z., Y.L., and B.C. contributed equally to this work. T.Z. carried out most of the experiments. T.Z. and Y.L. conceived, designed, and led the study, analyzed data, and wrote the manuscript. B.C. constructed the mathematical model, analyzed data, and wrote the manuscript. Z.X. contributed to mathematical model construction and M.L. designed the study and analyzed data. J.F., Y.Y., P.J., and L.G. contribute to in vivo experiment and data analysis. Y.B. collected and analyzed the clinical samples. F.X. and H.G. conceived, designed, and led the study, analyzed data, supervised research, and edited the manuscript. All authors discussed and commented on the manuscript.

## Supporting information



Supporting Information

## Data Availability

The raw data of RNA‐sequencing transcriptome have been deposited to the National Center for Biotechnology Information (NCBI) under the BioProject number PRJNA1263671.
