# Supplementary material for: Piezo1 Activation Improves NSCLC Liver Metastasis Immunotherapy by Overriding Matrix Stiffness‐Mediated Bimodal PD‐L1/CXCL10 Regulation
Source: Adv Sci (Weinh). 2025 Jun 29;12(32):e01335. doi: 10.1002/advs.202501335 (PMC12407327; doi:10.1002/advs.202501335)
Supplement: Supplementary file 1 — Supporting Information [file ADVS-12-e01335-s001.docx]

**Supporting Information for**

**Piezo1 Activation Improves NSCLC Liver Metastasis Immunotherapy by Overriding Matrix Stiffness-Mediated Bimodal PD-L1/CXCL10 Regulation**

Tian Zhang^a,b,c#^, Yuan Li^b,c#^, Bo Cheng^b,c#^, Zhao Xu^b,c^, Mengjie Liu^b,c,d^, Jinteng Feng^b,c,e^, Yixue Bai^a,b,c^, Yang Yu^a,b,c^, Panpan Jiang ^a,b,c^, Luying Geng ^a,b,c^, Feng Xu^b,c*^, Hui Guo^a,f*^

*^a^ Department of Medical Oncology, The Second Affiliated Hospital of Xi’an Jiaotong University, Xi’an 710061, P.R. China*

*^b^ The Key Laboratory of Biomedical Information Engineering of Ministry of Education, Xi’an Jiaotong University, Xi'an 710049, P.R. China*

*^c^ Bioinspired Engineering and Biomechanics Center (BEBC), Xi’an Jiaotong University, Xi'an 710049, P.R. China*

*^d^ Phase I Clinical Trial Ward, The Second Affiliated Hospital of Xi'an Jiaotong University, Xi'an 710049, P.R. China*

*^e^ Department of Thoracic Surgery, The First Affiliated Hospital of Xi’an Jiaotong University, Xi’an 710061, P.R. China*

*^f^* *The Key Laboratory of Surgical Critical Care and Life Support of Ministry of Education, Xi’an Jiaotong University, Xi'an 710049, P.R. China*

^#^ Authors contributed equally

*Corresponding authors: Feng Xu, Hui Guo

**Email:** fengxu@mail.xjtu.edu.cn; guohui@xjtufh.edu.cn

**
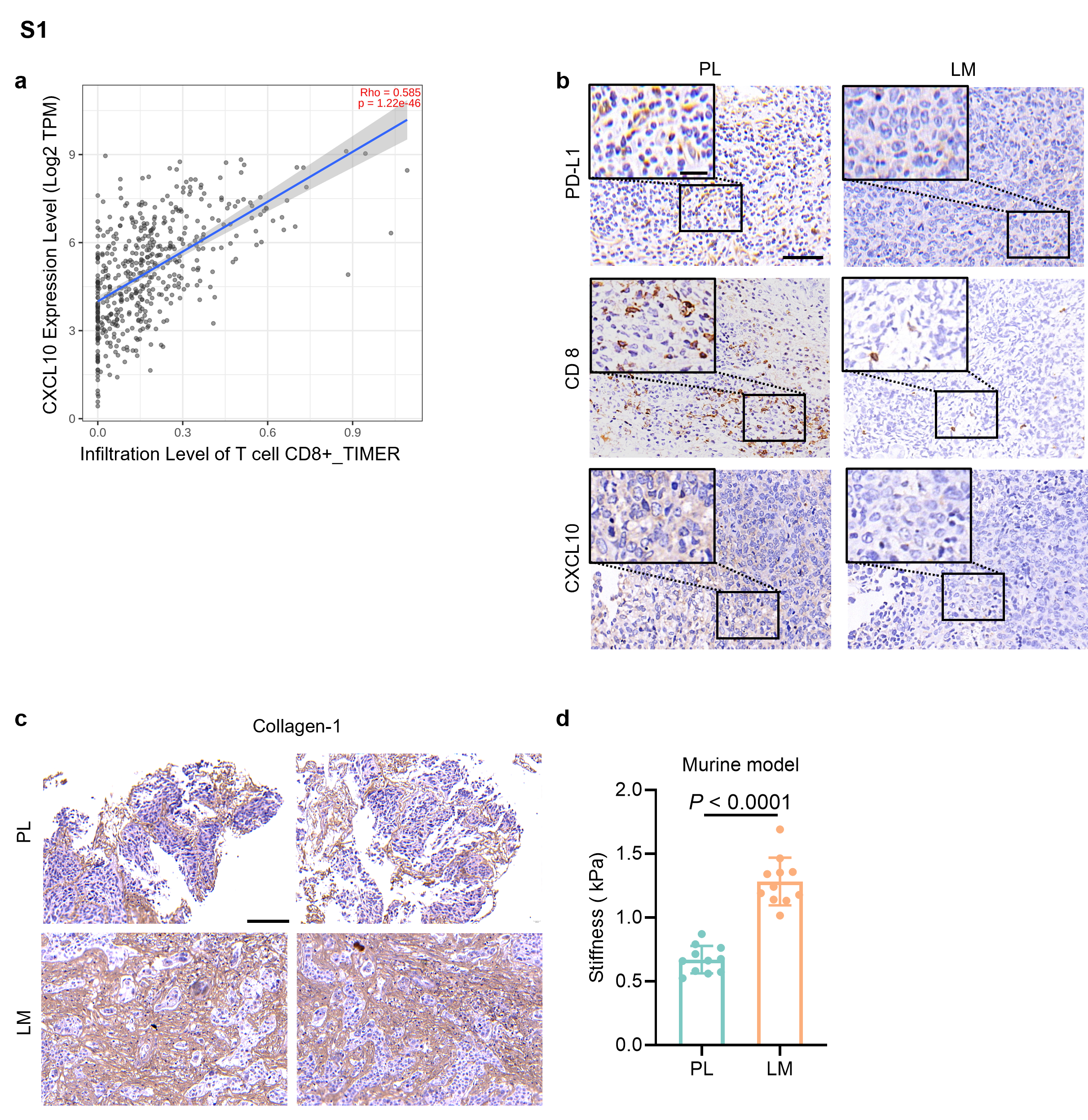
**

**Figure S1. The organ-specific microenvironments of PL and LM sites. a**, The expression of CXCL10 in NSCLC tissues is positively correlated with CD8^+^ T cell infiltration, according to the TIMER database. **b_,_** The PD-L1 expression, CD8^+^ T cell and CXCL10 IHC staining of PL and LM sites in murine models. The scale bars indicate 20 μm (enlarged image in upper left corner) and 60 μm, respectively. **c_,_** The Collagen-1 IHC staining of PL and LM sites in patients’ tissues. The scale bar indicates 120 μm. **d_,_** The Young’s modulus of PL sites and LM sites in the murine models as measured by BOSE ELECTROFORCE 3200 (for both PL sites and LM sites tissues, N = 11). Data are compared by a two-tailed Student’s *t*-test (**d)**. In (**d**), data are shown as mean ± S.E.M. CXCL10, chemokine C-X-C ligand 10; NSCLC, non-small cell lung cancer; PL, primary lung cancer; LM, liver metastasis; TIMER, Tumor Immune Estimation Resource; IHC, immunohistochemistry.


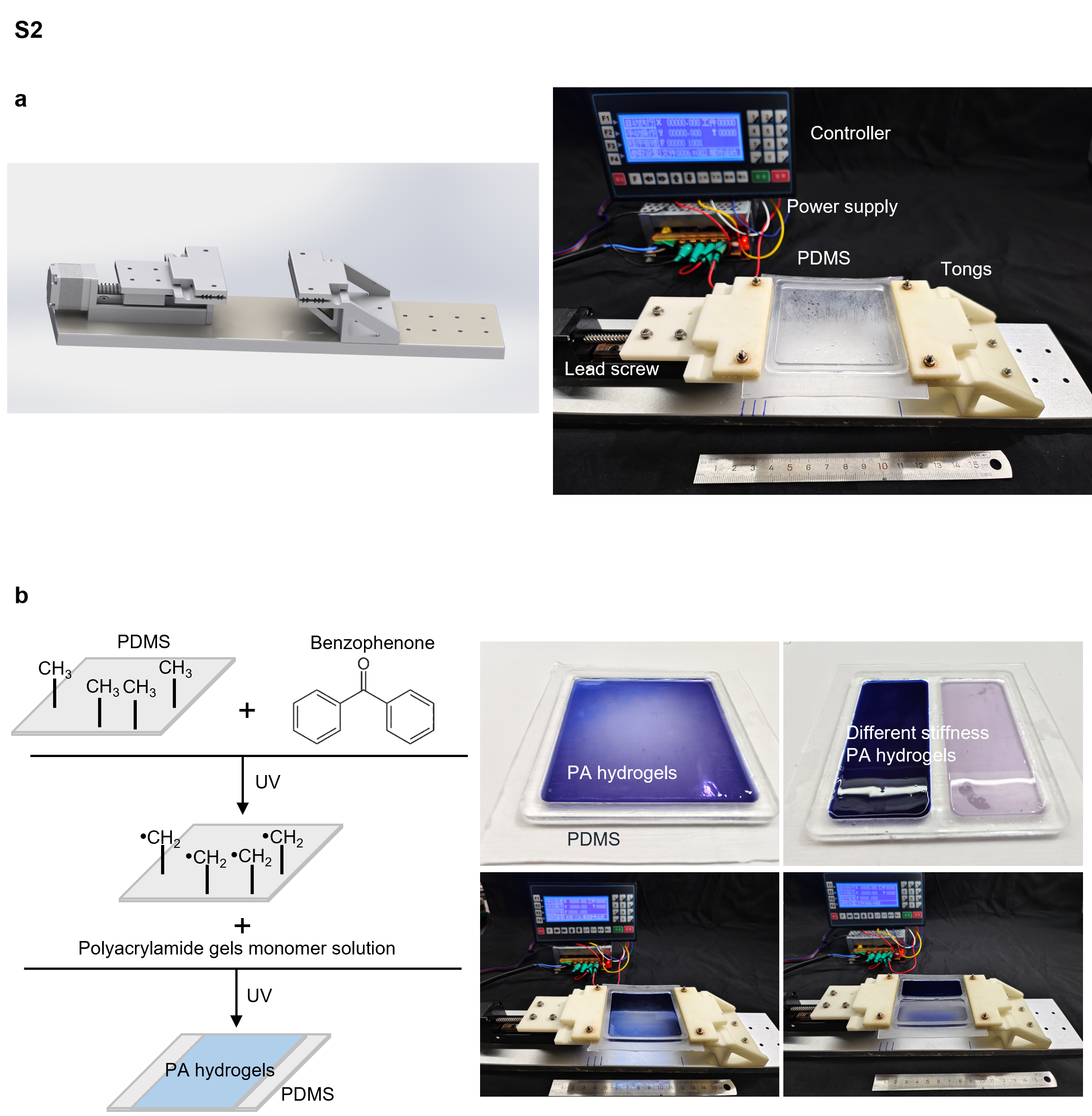


**Figure S2. Custom-designed stretching platform. a_,_** The stretching platform. **b_,_** The approach used to create hydrogel coatings on a PDMS surface. PDMS, polydimethylsiloxane.

**
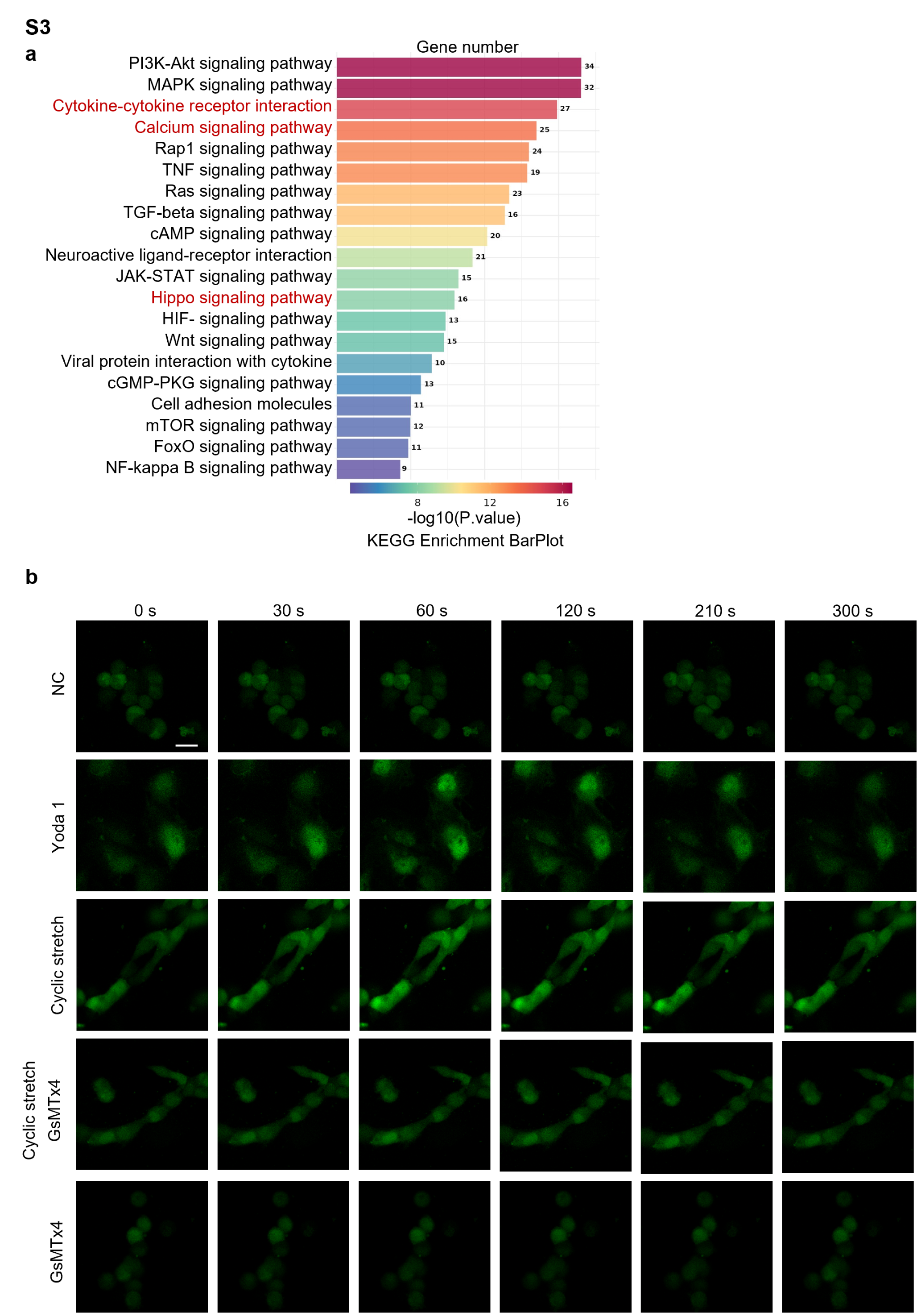
**

**Figure S3. Cyclic stretch triggers the calcium signaling pathway via the Piezo1 in NSCLC cells. a_,_** KEGG enrichment analysis of environmental information processing-related pathways. Data are obtained from transcriptome sequencing of H1299 cells cultured on static or cyclically stretched PDMS. **b_,_** The immunofluorescence assay of calcium ion fluorescent probes. The scale bar indicates 20 μm. KEGG, Kyoto Encyclopedia of Genes and Genomes; PDMS, polydimethylsiloxane.


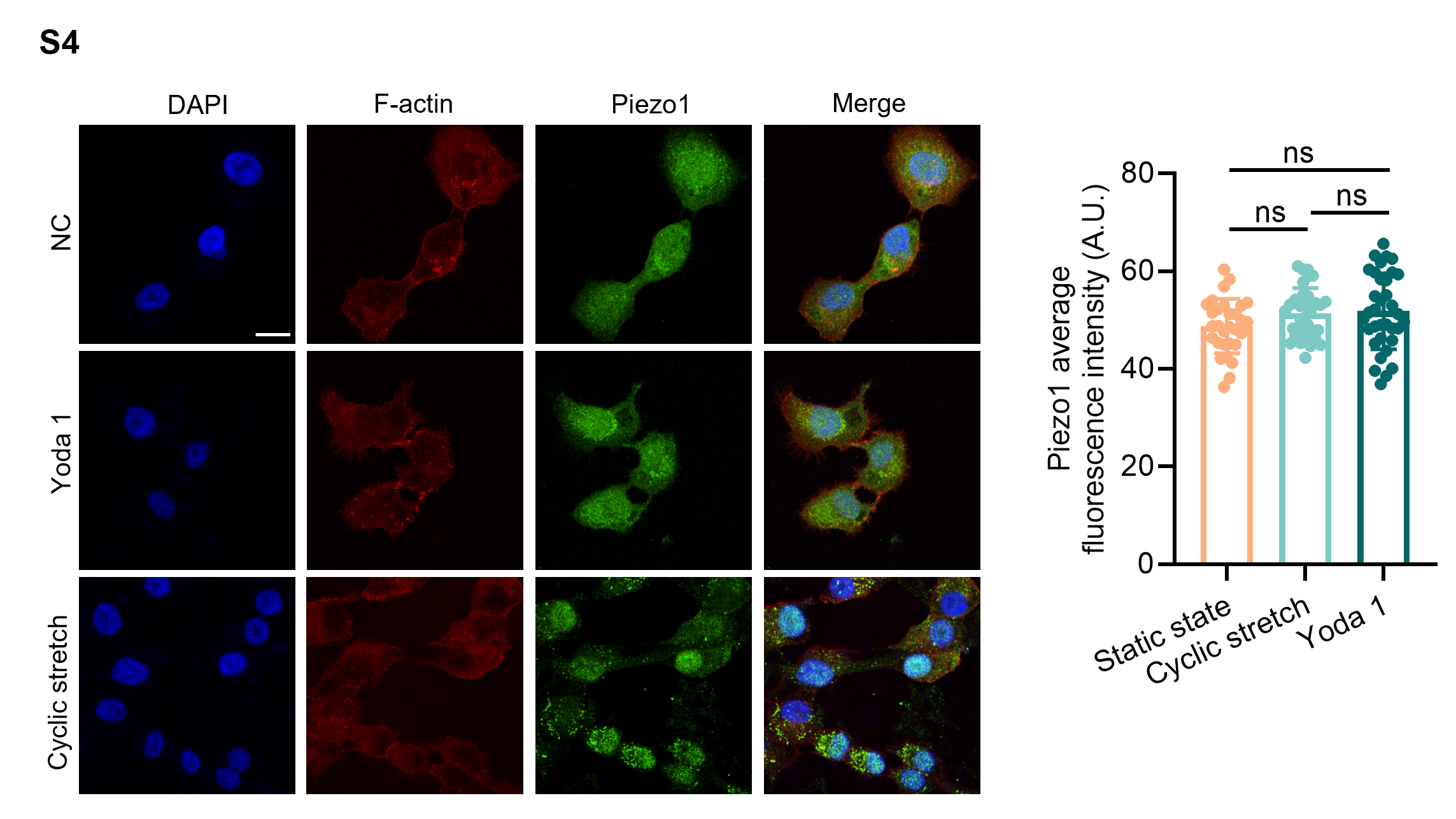


**Figure S4. Piezo1 activation barely alters Piezo1 expression.** The immunofluorescence assay and the quantification of the immunofluorescence of Piezo1 (N ≥ 5, n ≥ 30 cells). Data are compared by a two-tailed Student’s *t*-test. All data are shown as mean ± S.E.M. N, the number of independent experiments. n, the number of cells counted. The scale bar indicates 20 μm. Yoda 1, Piezo1-specific agonist; GsMTx4, Piezo 1 ion channel-specific inhibitor.


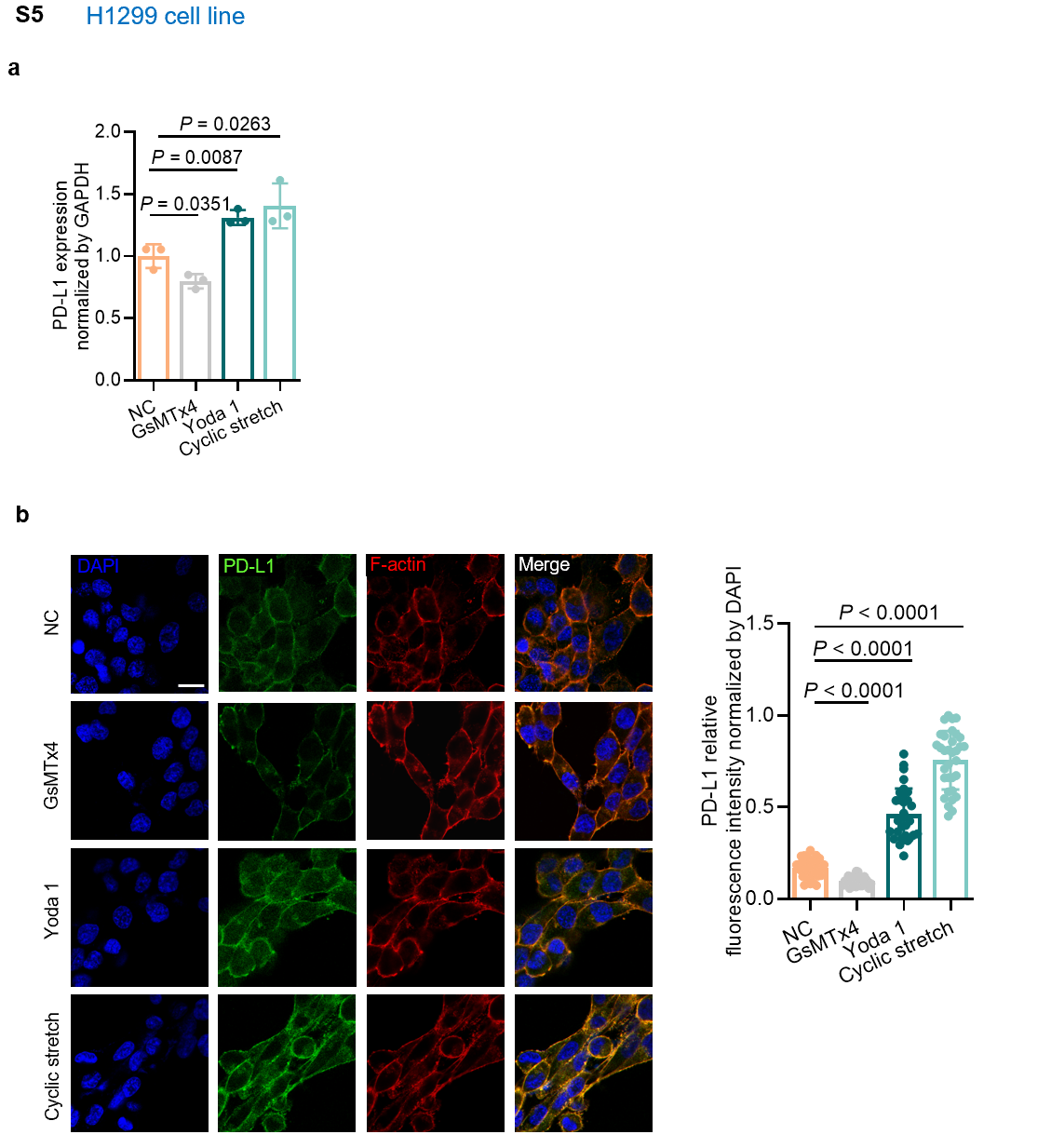


**Figure S5.** **Cyclic stretch raises the expression of PD-L1 by Piezo1 activity in H1299 cells. a**, Quantification of the western blotting of the level of PD-L1 in H1299 cells with 24-hr treatment of GsMTx4, Yoda 1, cyclic stretch, static condition. PD-L1 protein levels were normalized to GAPDH (N = 3). **b**, The immunofluorescence assay and the quantification of the immunofluorescence of PD-L1 in H1299 cells with 24-hr treatment of GsMTx4, Yoda 1, cyclic stretch, static condition (N ≥ 5, n ≥ 30 cells). Data are compared by a two-tailed Student’s *t*-test (**a-b**). All data are shown as mean ± S.E.M. N, the number of independent experiments. Yoda 1, Piezo1-specific agonist; GsMTx4, Piezo 1 ion channel-specific inhibitor.


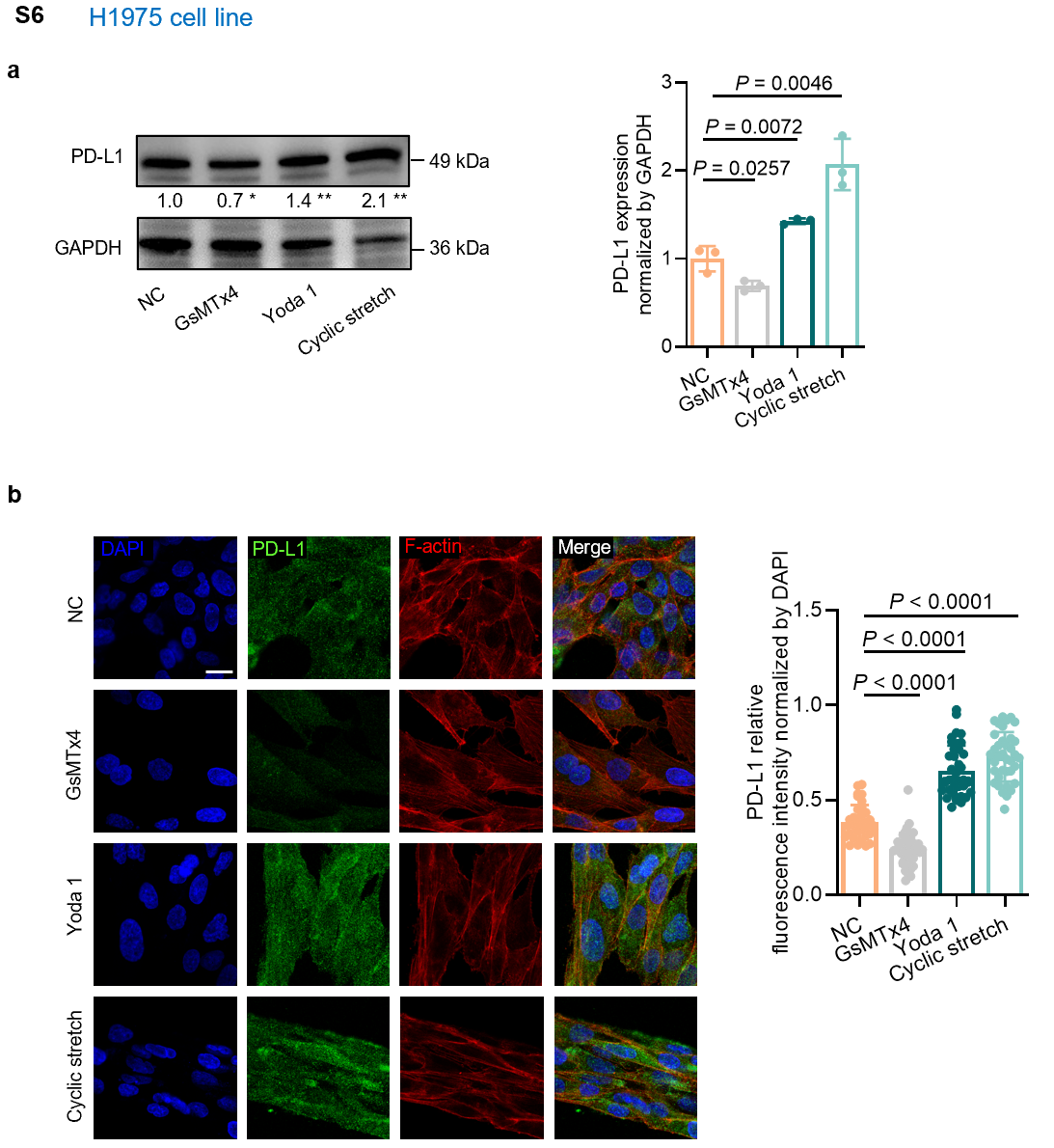


**Figure S6.** **Cyclic stretch raises the expression of PD-L1 by Piezo1 activity in H1975 cells. a**, The western blotting of the level of PD-L1 in H1975 cells with 24-hr treatment of GsMTx4, Yoda 1, cyclic stretch, static condition. PD-L1 protein levels were normalized to GAPDH (N = 3). **b**, The immunofluorescence assay and the quantification of the immunofluorescence of PD-L1 in H1975 cells with 24-hr treatment of GsMTx4, Yoda 1, cyclic stretch, static condition (N ≥ 5, n ≥ 30 cells). Data are compared by a two-tailed Student’s *t*-test (**a-b**). In (**a-b**), data are shown as mean ± S.E.M. N, the number of independent experiments. n, the number of cells counted. Yoda 1, Piezo1-specific agonist; GsMTx4, Piezo 1 ion channel-specific inhibitor.


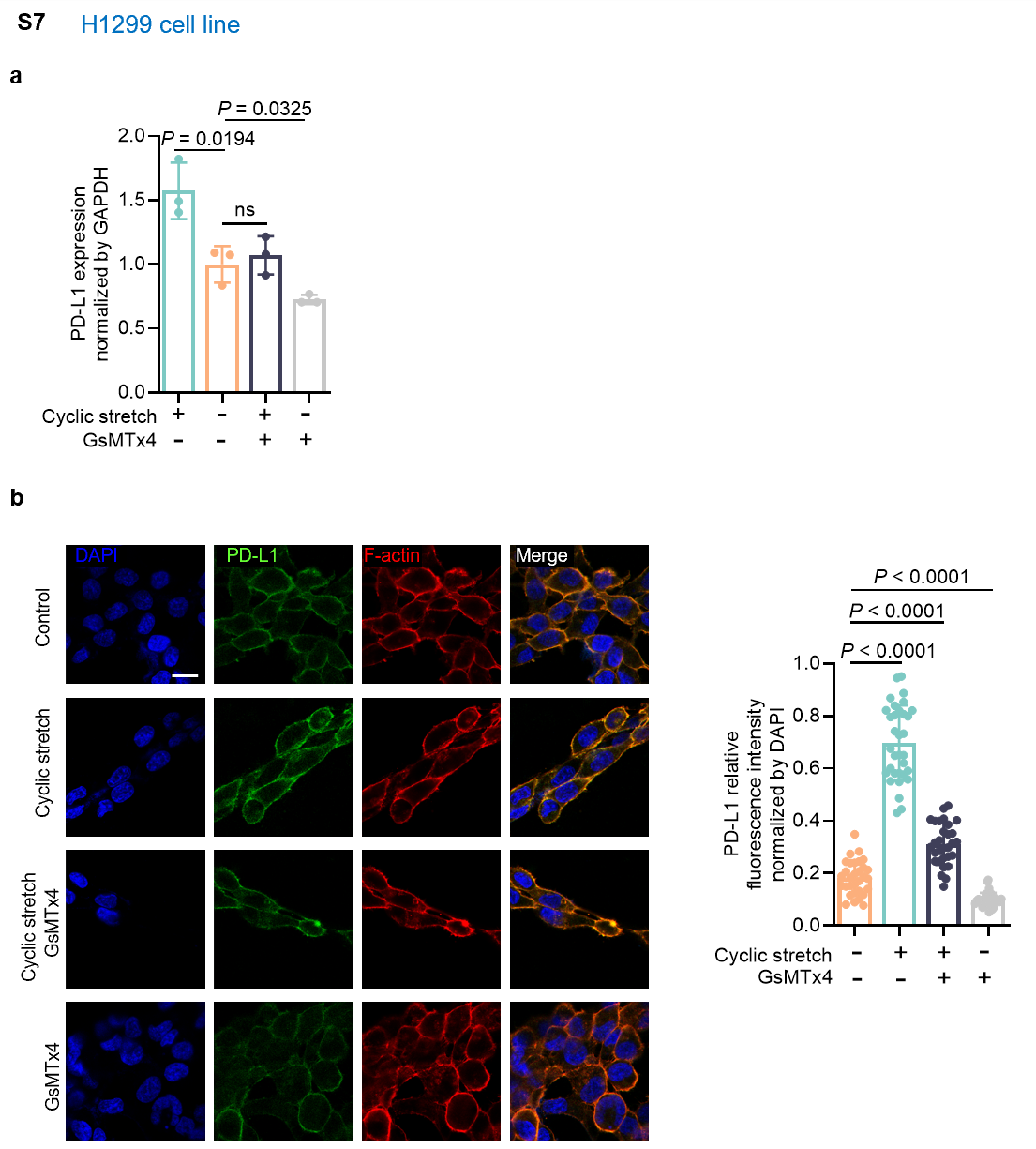


**Figure S7.** **PD-L1 expression is dependent on the activation of Piezo1 in H1299 cells. a.** Quantification of the western blotting of the level of PD-L1 in H1299 cells with 24-hr treatment of GsMTx4, cyclic stretch, static condition, and 24-hr cyclic stretch with simultaneous treatment of GsMTx4. PD-L1 protein levels were normalized to GAPDH (N = 3). **b.** The immunofluorescence assay and the quantification of the immunofluorescence of PD-L1 in H1299 cells with 24-hr treatment of GsMTx4, cyclic stretch, static condition, and 24-hr cyclic stretch with simultaneous treatment of GsMTx4 (N ≥ 5, n ≥ 30 cells). Data are compared by a two-tailed Student’s *t*-test (**a-b**). In (**a-b**), data are shown as mean ± S.E.M. N, the number of independent experiments. n, the number of cells counted. Yoda 1, Piezo1-specific agonist; GsMTx4, Piezo 1 ion channel-specific inhibitor.


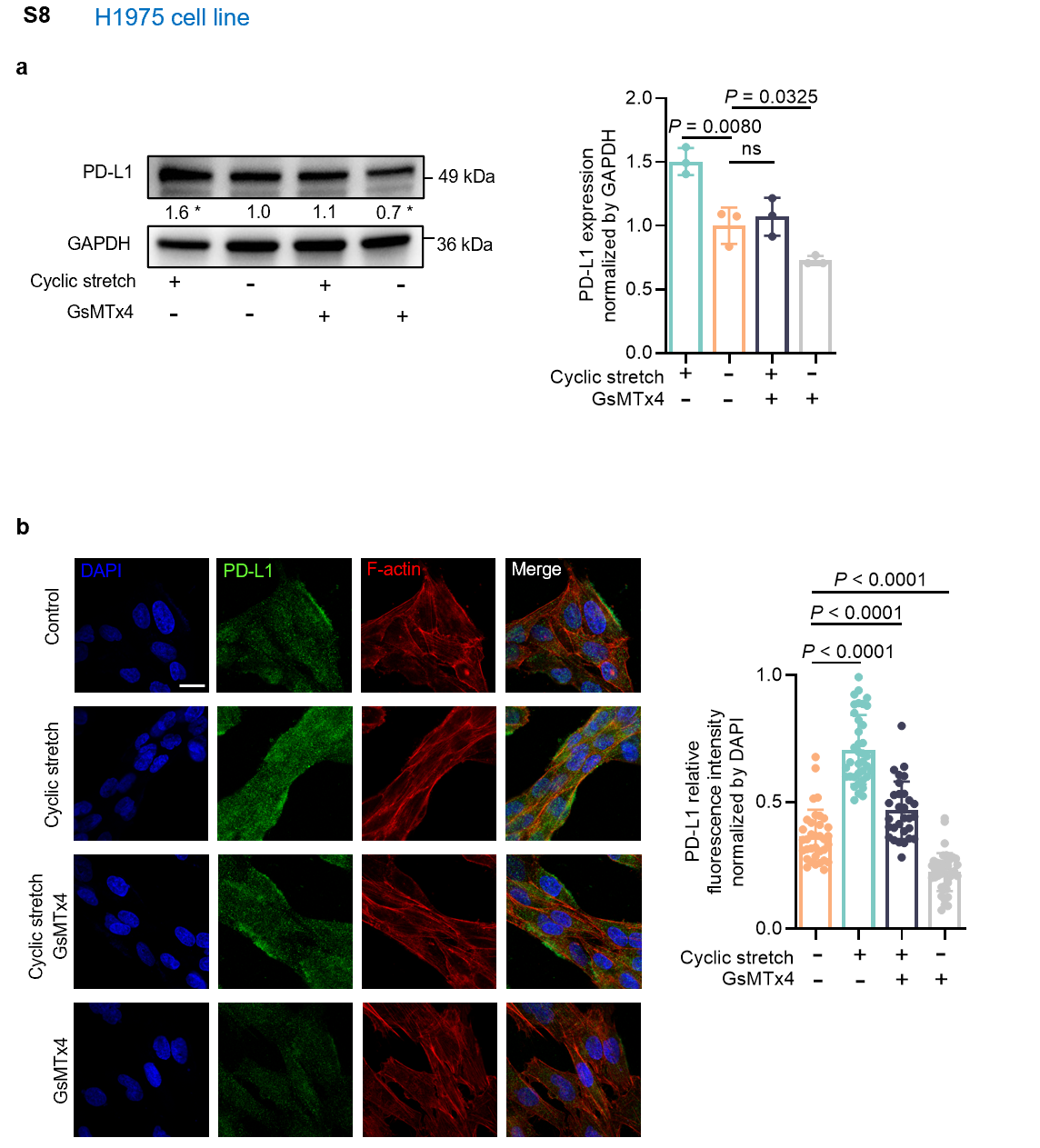


**Figure S8.** **Piezo1 inhibition suppresses the stretch-induced PD-L1 upregulation in H1975 cells. a**, The western blotting of the level of PD-L1 in H1975 cells on static and cyclical stretch condition with/without GsMTx4 treatment. PD-L1 protein levels were normalized to GAPDH (N = 3). **b**, The immunofluorescence assay and the quantification of the immunofluorescence of PD-L1 in H1975 cells with 24-hr treatment of GsMTx4, cyclic stretch, static condition, and 24-hr cyclic stretch with simultaneous treatment of GsMTx4 (N ≥ 5, n ≥ 30 cells). Data are compared by a two-tailed Student’s *t*-test (**a-b**). In (**a-b**), data are shown as mean ± S.E.M. N, the number of independent experiments. n, the number of cells counted. GsMTx4, Piezo 1 ion channel-specific inhibitor.


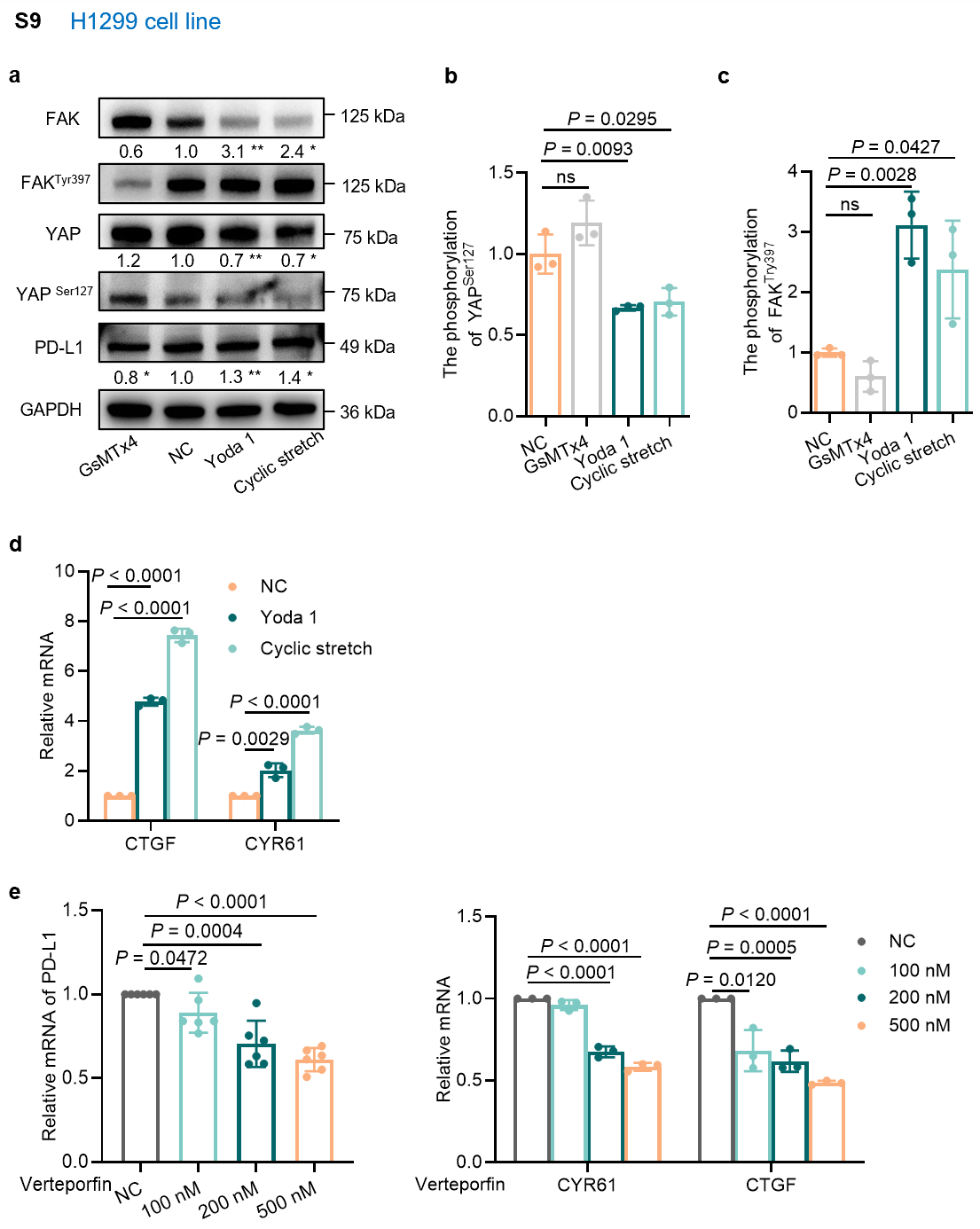


**Figure S9.** **Piezo1 activity regulates the expression of PD-L1 through YAP pathway. a**, The western blotting of the phosphorylation degree of YAP^Ser127^ and FAK^Tyr397^ in H1299 cells with treatment of GsMTx4, Yoda 1, cyclic stretch, static condition (N = 3). **b**, Quantification of the phosphorylation degree of YAP^Ser127^ in H1299 cells with treatment of GsMTx4, Yoda 1, cyclic stretch, static condition. Degree of YAP^Ser127^ phosphorylation = gray value of YAP^Ser127^ / gray value of YAP (N = 3). **c**, Quantification of the phosphorylation degree of FAK^Tyr397^ in H1299 cells with 24-hr treatment of GsMTx4, Yoda 1, cyclic stretch, static condition. Degree of FAK^Tyr397^ phosphorylation = gray value of FAK^Tyr397^ / gray value of FAK (N = 3). **d**, The CYR61 and CTGF mRNA levels in H1299 cells with treatment of Yoda 1, cyclic stretch, static condition (N = 3). **e**, The CYR61, CTGF and PD-L1, mRNA levels in H1299 cells with treatment of Verteporfin (N = 3). Data are compared by a two-tailed Student’s *t*-test (**b-e**). All data are shown as mean ± S.E.M. N, the number of independent experiments. Yoda 1, Piezo1-specific agonist; GsMTx4, Piezo 1 ion channel-specific inhibitor; Verteporfin, YAP transcriptional function inhibitor.


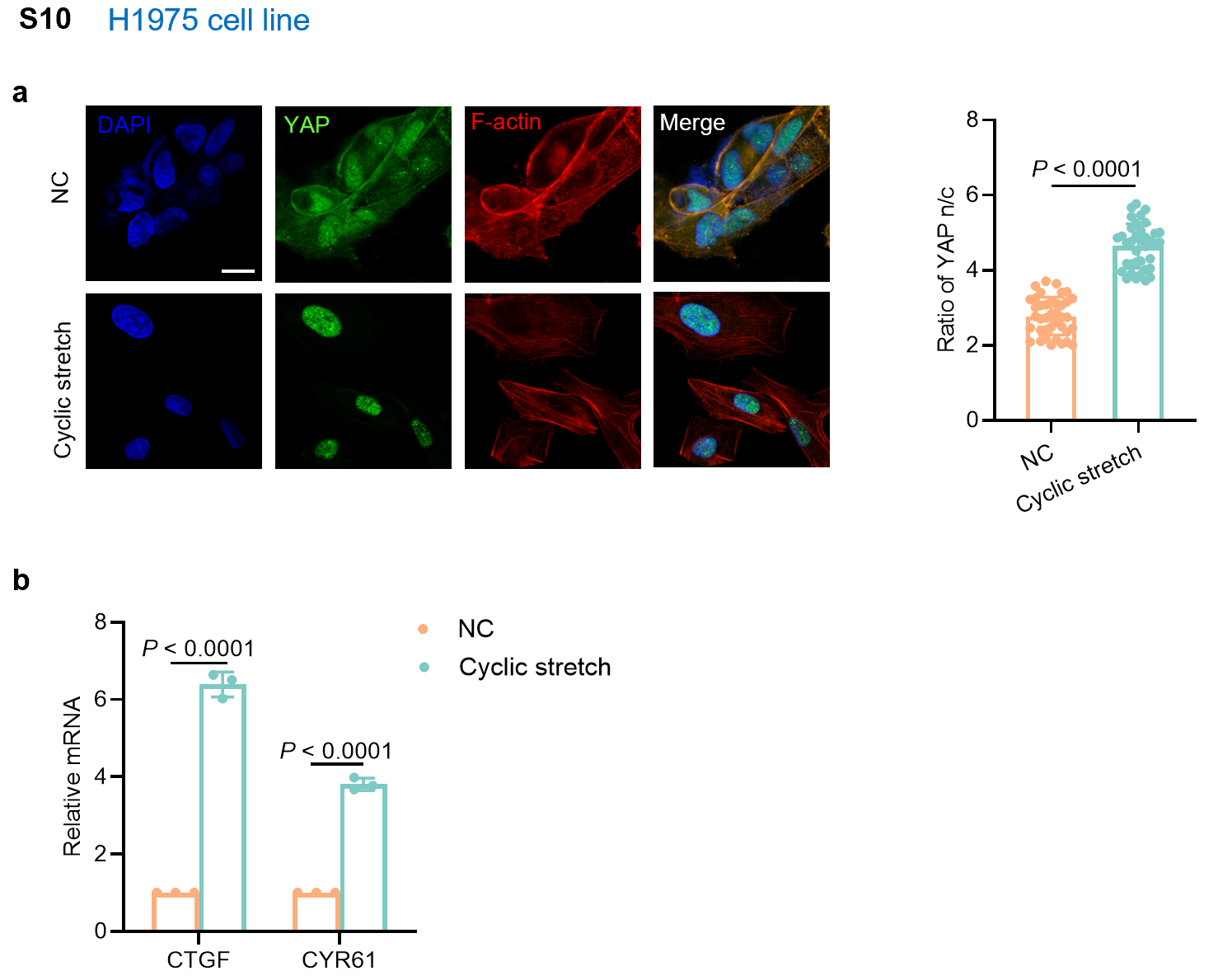


**Figure S10. Cyclic stretch upregulates the expression of PD-L1 via YAP-mediated Piezo1 activation in H1975 cells. a.** The immunofluorescent images of YAP and quantification of YAP nuclear/cytoplasmic (n/c) ratio. (N ≥ 5, n ≥ 35 cells. The scale bars indicate 10 μm). **b,** The CYR61 and CTGF mRNA levels in H1975 cells with treatment of cyclic stretch, static condition (N = 3). Data are compared by a two-tailed Student’s *t*-test. In (**a-b**), all data are shown as mean ± S.E.M.


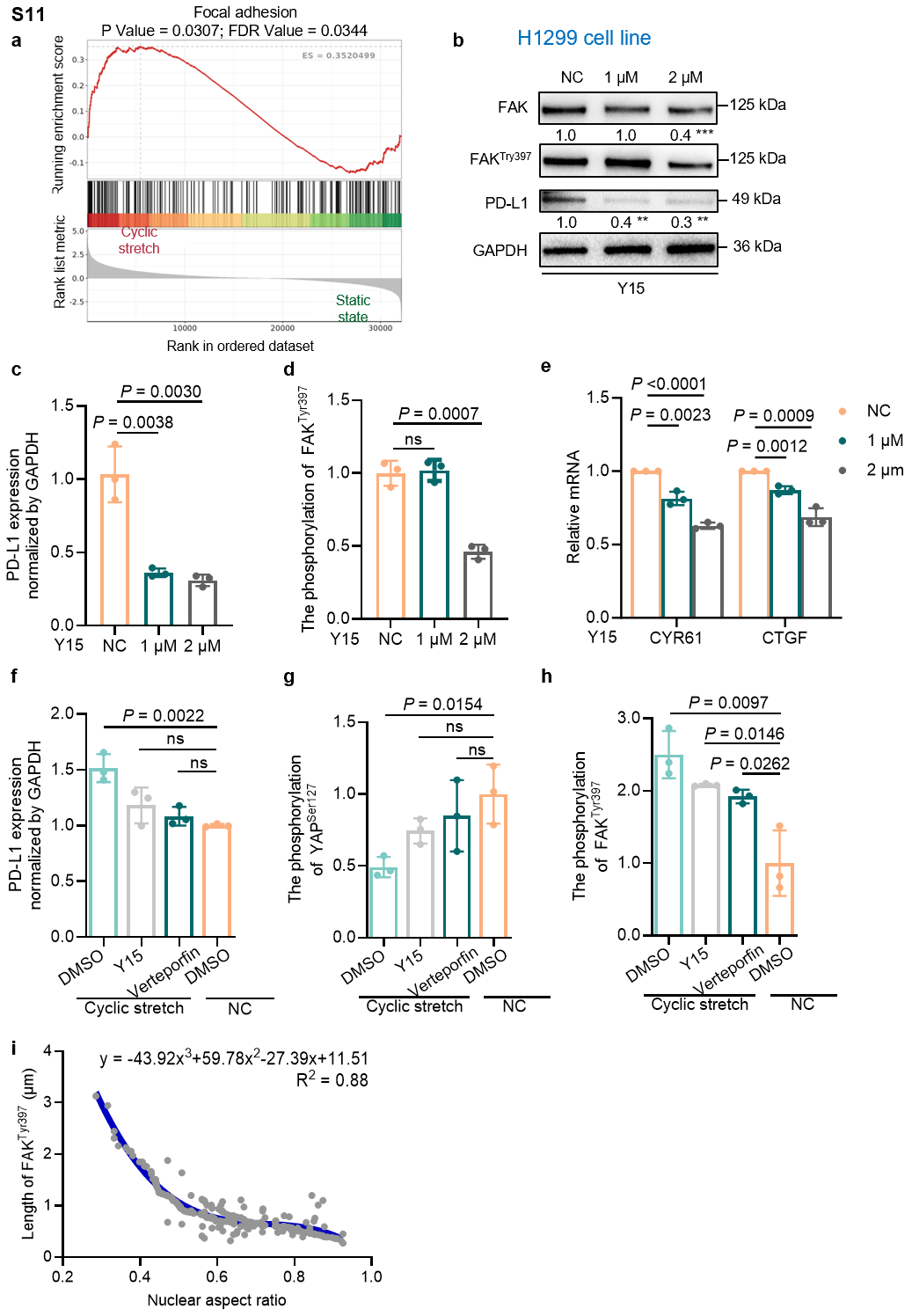


**Figure S11.** **FAK phosphorylation participates in the regulation of PD-L1 by Piezo1. a**, KEGG enrichment of focal adhesion. Data are obtained from transcriptome sequencing in H1299 cells cultured on static PDMS or cyclically stretched PDMS. **b**, The western blotting of the PD-L1 level and the phosphorylation degree of FAK^Tyr397^ in H1299 cells with treatment of Y15 (N = 3). **c**, Quantification of the level of PD-L1 in H1299 cells with treatment of Y15. Protein expressions were normalized by GAPDH (N = 3). **d**, Quantification of the phosphorylation of FAK^Tyr397^ in H1299 cells with treatment of Y15. Degree of FAK^Tyr397^ phosphorylation = gray value of FAK^Tyr397^ / gray value of FAK (N = 3). **e**, The CYR61 and CTGF mRNA level in H1299 cells treated by Y15 (N = 3). **f,** Quantification of the level of PD-L1 in H1299 cells treated with Verteporfin and Y15 in the condition of cyclic stretch and static state. Protein expressions were normalized by GAPDH (N = 3). **g,** Quantification of the phosphorylation of YAP^Ser127^ in H1299 cells treated with Verteporfin and Y15 in the condition of cyclic stretch and static state. Degree of YAP^Ser127^ phosphorylation = gray value of YAP^Ser127^ / gray value of YAP (N = 3). **h,** Quantification of the phosphorylation degree of FAK^Tyr397^ in H1299 cells treated with Verteporfin and Y15 in the condition of cyclic stretch and static state. Degree of FAK^Tyr397^ phosphorylation = gray value of FAK^Tyr397^ / gray value of FAK (N = 3). **i,** Linear analysis was performed based on the length of FAK^Tyr397^ and nuclear aspect ratio. Data are compared by a two-tailed Student’s *t*-test (**c-h**). In (**c-h**), data are shown as mean ± S.E.M. N, the number of independent experiments. KEGG, Kyoto Encyclopedia of Genes and Genomes; PDMS, polydimethylsiloxane; Y15, Phosphorylation inhibitor of FAK^Tyr397^.


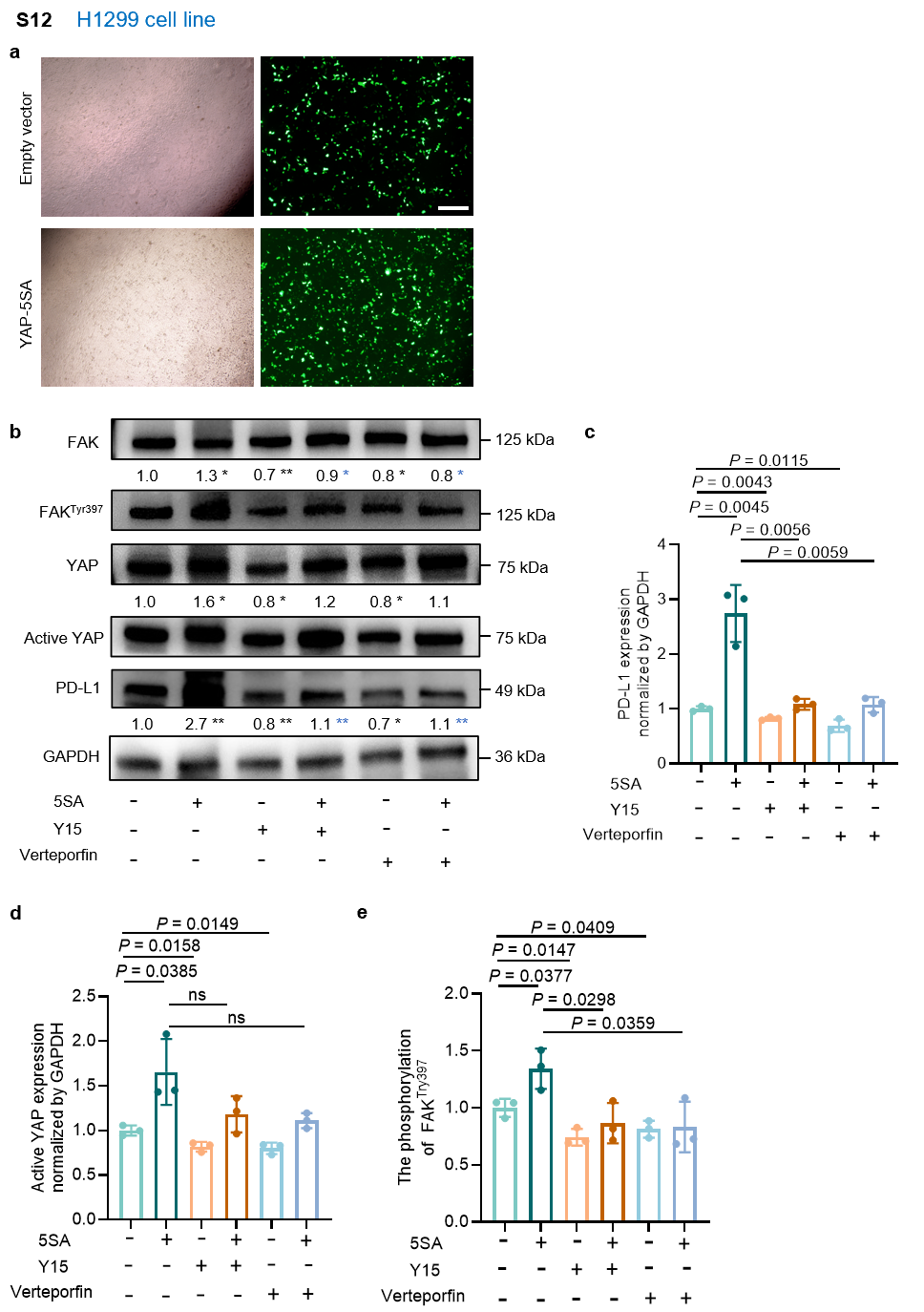


**Figure S12.** **Overexpression of active YAP rescues the downregulation of PD-L1 due to FAK and YAP inhibition. a**, Construction of active YAP (5SA) overexpression H1299 cells. **b**, The western blotting of the PD-L1 and active YAP level, and the phosphorylation degree of FAK^Tyr397^ in control H1299 cells and YAP-5SA H1299 cells treated by Y15 and Verteporfin (N = 3). *, The comparison between the empty vector group and the empty vector group treated with Y15 or Verteporfin. (*, dark blue, between the 5SA-YAP group and the 5SA-YAP group treated with Y15 or Verteporfin).**c**, Quantification of the level of PD-L1 in control and YAP-5SA H1299 cells treated by Y15 and Verteporfin. Protein expressions were normalized by GAPDH (N = 3). **d**, Quantification of the level of active YAP in control H1299 cells and YAP-5SA H1299 cells treated by Y15 and Verteporfin. Protein expressions were normalized by GAPDH (N = 3). **e**, Quantification of the phosphorylation of FAK^Tyr397^ in control and YAP-5SA H1299 cells treated by Y15 and Verteporfin. Degree of FAK^Tyr397^ phosphorylation = gray value of FAK^Tyr397^ / gray value of FAK (N = 3). Data are compared by a two-tailed Student’s *t*-test (**c-e**). All data are shown as mean ± S.E.M. N, the number of independent experiments. 5SA, the plasmid of overexpression of constitutively active YAP; Verteporfin, YAP transcriptional function inhibitor; Y15, Phosphorylation inhibitor of FAK^Tyr397^.


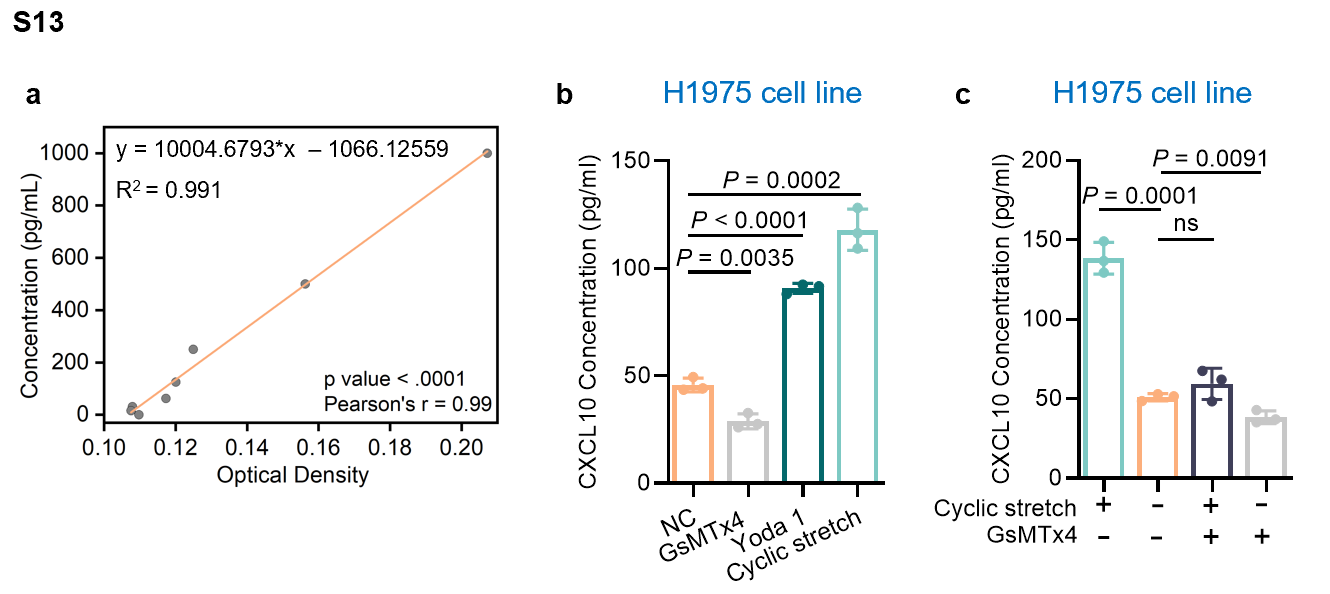


**Figure S13.** **Piezo1 activation raises the secretion of chemokines CXCL10 in H1975 cells. a**, Standard curve of CXCL10 secretion as measured by ELISA kit. **b**, The expression levels of CXCL10 in the supernatant of H1975 cells as determined by ELISA after 24-hr treatment of GsMTx4, Yoda 1, cyclic stretch, static condition (N = 3). **c**, The expression levels of CXCL10 in the supernatant of H1975 cells as determined by ELISA after24-hr treatment of GsMTx4, cyclic stretch, static condition, and 24-hr cyclic stretch with simultaneous treatment of GsMTx4 (N = 3). Data are compared by a two-tailed Student’s *t*-test (**b-c**). All data are shown as mean ± S.E.M. N, the number of independent experiments. CXCL10, chemokine C-X-C ligand 10; ELISA, enzyme-linked immunosorbnent assay. Yoda 1, Piezo1-specific agonist; GsMTx4, Piezo 1 ion channel-specific inhibitor.


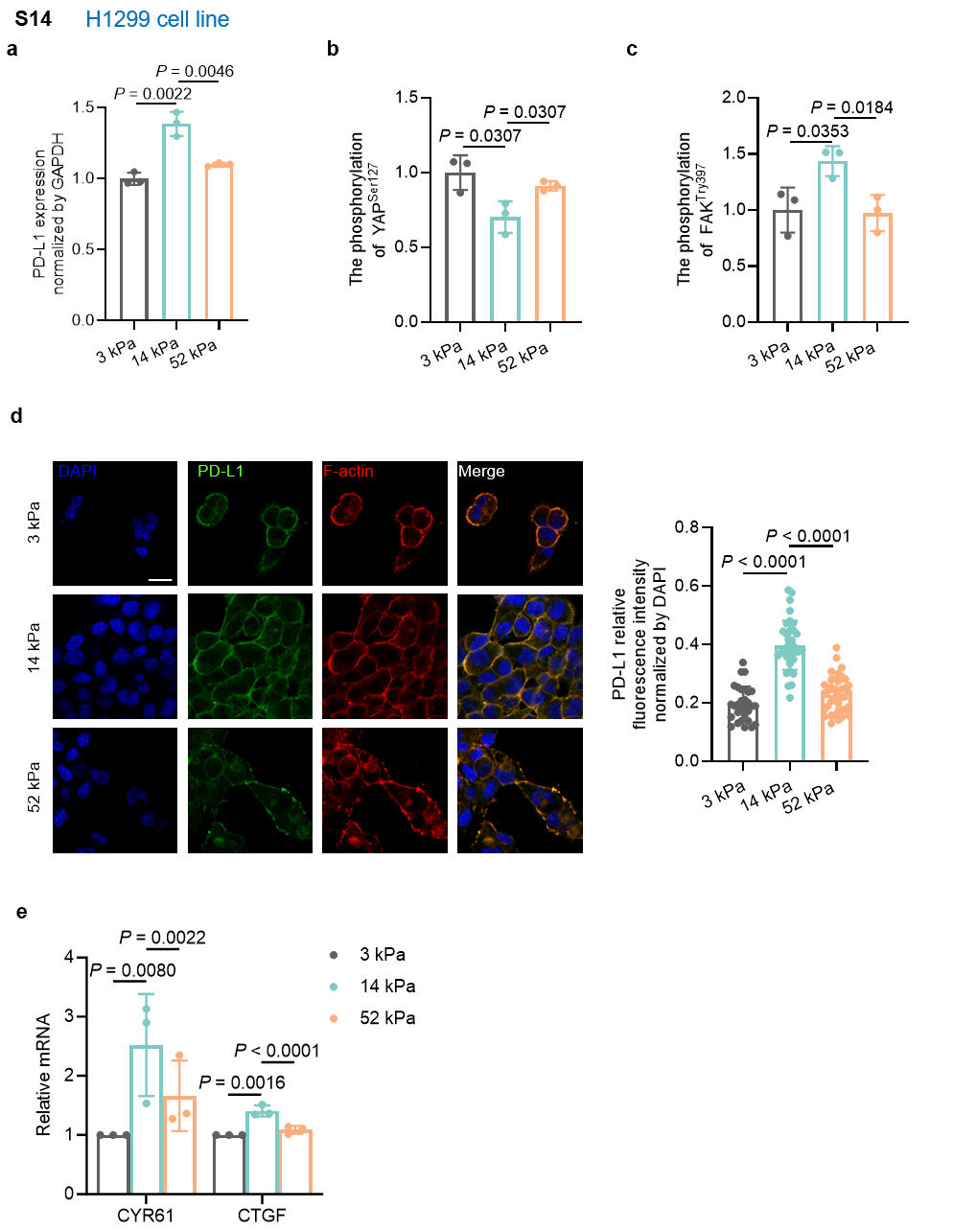


**Figure S14.** **ECM with 14kPa stiffness owns the optimal regulation of PD-L1.** **a**, Quantification of the PD-L1 level in H1299 cells cultured on 3 kPa, 14 kPa, and 52 kPa hydrogels. Protein expressions were normalized by GAPDH (N = 3). **b**, Quantification of the phosphorylation of YAP^Ser127^ in H1299 cells cultured on 3 kPa, 14 kPa, and 52 kPa hydrogels. Degree of YAP^Ser127^ phosphorylation = gray value of YAP^Ser127^ / gray value of YAP (N = 3). **c**, Quantification of the phosphorylation of FAK^Tyr397^ in H1299 cells cultured on 3 kPa, 14 kPa, and 52 kPa hydrogels. Degree of FAK^Tyr397^ phosphorylation = gray value of FAK^Tyr397^ / gray value of FAK (N = 3). **d**, The immunofluorescence assay and the quantification of the immunofluorescence of PD-L1 in H1299 cells cultured on 3 kPa, 14 kPa, and 52 kPa hydrogels (N ≥ 5, n ≥ 30 cells). **e**, The CYR61 and CTGF mRNA levels in H1299 cells cultured on 3 kPa, 14 kPa, and 52 kPa hydrogels (N = 3). Data are compared by a two-tailed Student’s *t*-test (**a-e**). In (**a-e**), all data are shown as mean ± S.E.M. N, the number of independent experiments. n, the number of cells counted. ECM, extracellular matrix.


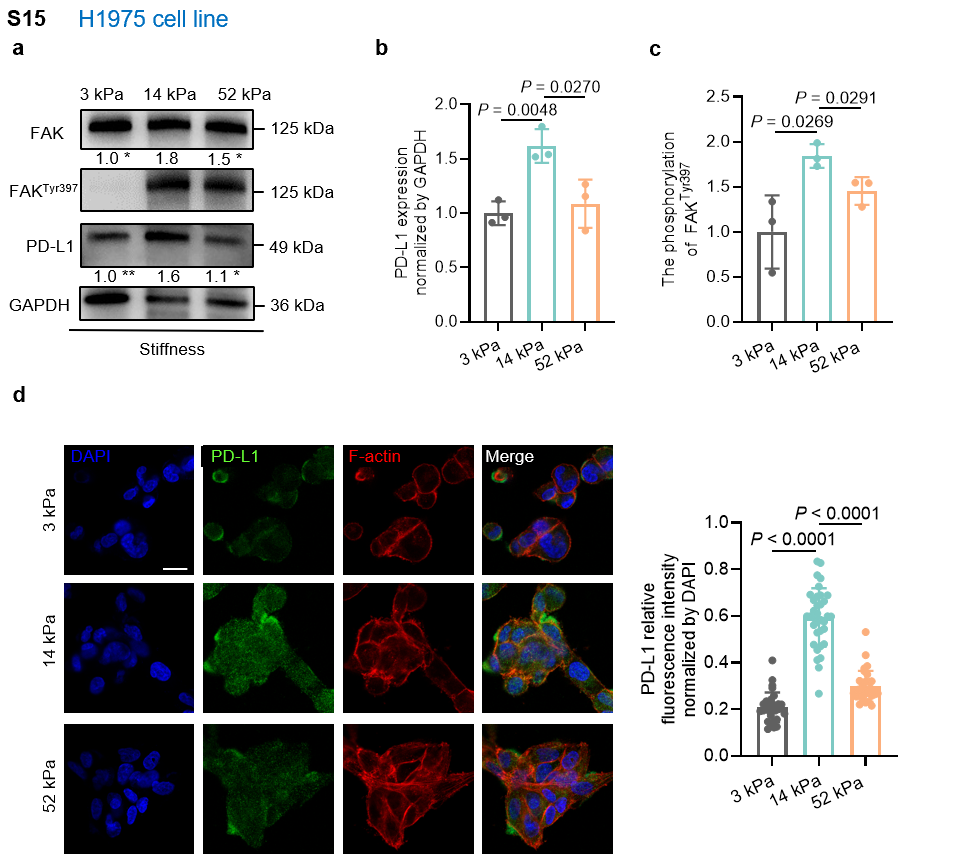


**Figure S15. ECM with 14kPa stiffness owns the optimal regulation of PD-L1 in H1975 cells. a**, The western blotting of PD-L1 and FAK^Tyr397^ in H1975 cells cultured on 3 kPa, 14 kPa, and 52 kPa hydrogels. (N = 3). **b**, Quantification of the PD-L1 in H1975 cells cultured on 3 kPa, 14 kPa, and 52 kPa hydrogels. Protein expressions were normalized by GAPDH (N = 3). **c**, Quantification of the phosphorylation of FAK^Tyr397^ in H1975 cells cultured on 3 kPa, 14 kPa, and 52 kPa hydrogels. Degree of FAK^Tyr397^ phosphorylation = gray value of FAK^Tyr397^ / gray value of FAK (N = 3). **d**, The immunofluorescence assay and the quantification of PD-L1 in H1975 cells cultured on 3 kPa, 14 kPa, and 52 kPa hydrogels (N ≥ 5, n ≥ 30 cells). Data are compared by a two-tailed Student’s *t*-test (**b-d**). In(**b-d**), all data are shown as mean ± S.E.M. N, the number of independent experiments. n, the number of cells counted. ECM, extracellular matrix.


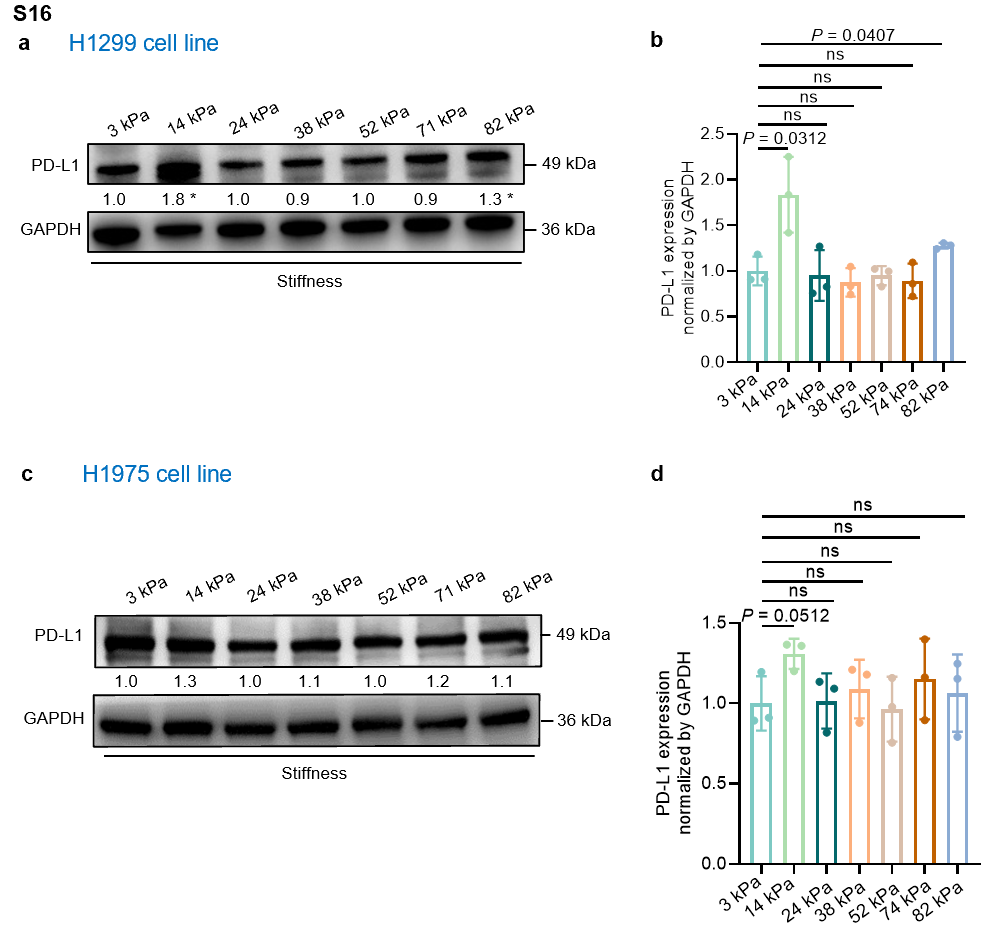


**Figure S16.** **Optimal stiffness exists in the regulation of PD-L1 by ECM. a**, The western blotting and quantification of the PD-L1 in H1299 cells cultured on a broader range of ECM stiffness. Protein expressions were normalized by GAPDH (N = 3). **b**, The western blotting and quantification of the PD-L1 in H1975 cells cultured on a broader range of ECM stiffness. Protein expressions were normalized by GAPDH (N = 3). Data are compared by a two-tailed Student’s *t*-test (**a-b**). In(**a-b**), all data are shown as mean ± S.E.M. N, the number of independent experiments. ECM, extracellular matrix.


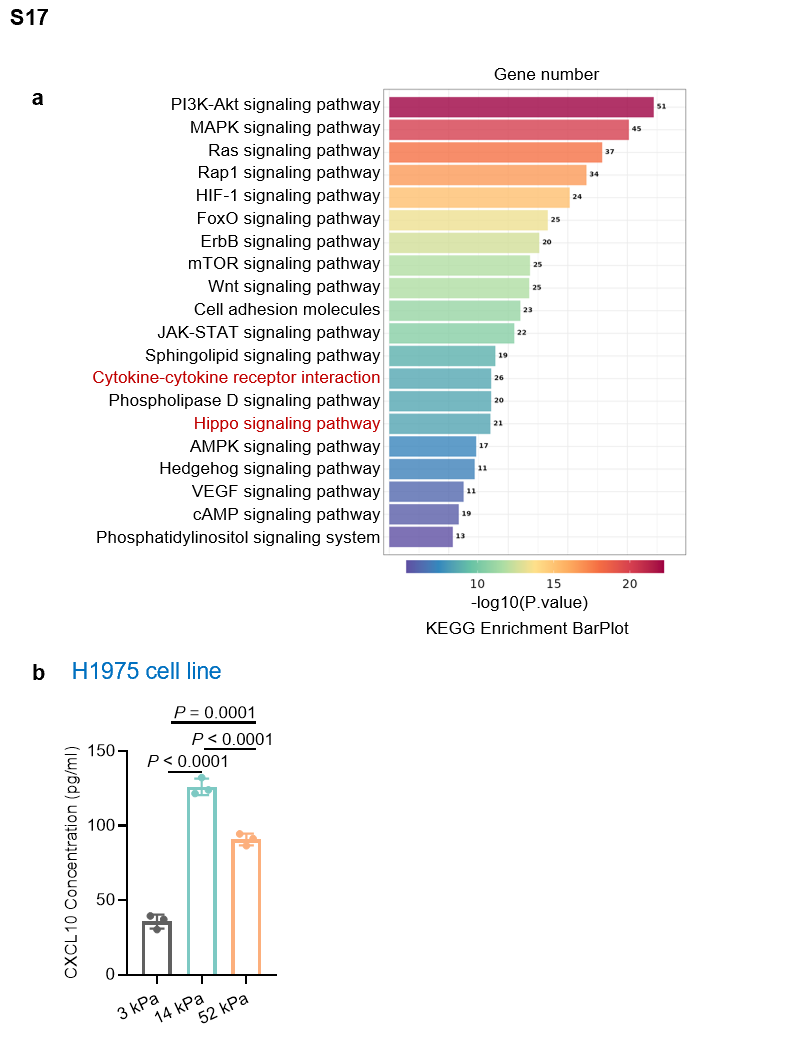


**Figure S17. ECM with 14kPa stiffness owns the optimal regulation of CXCL10. a**, KEGG enrichment analysis of environmental information processing-related pathways. Data are obtained from transcriptome sequencing in H1299 cells cultured on 3 kPa, 14 kPa, and 52 kPa hydrogels. **b**, The expression levels of CXCL10 in the supernatant of H1975 cells cultured on 3 kPa, 14 kPa, and 52 kPa hydrogels (N = 3). Data are compared by a two-tailed Student’s *t*-test (**b**). All data are shown as mean ± S.E.M. N, the number of independent experiments. CXCL10, chemokine C-X-C ligand 10.


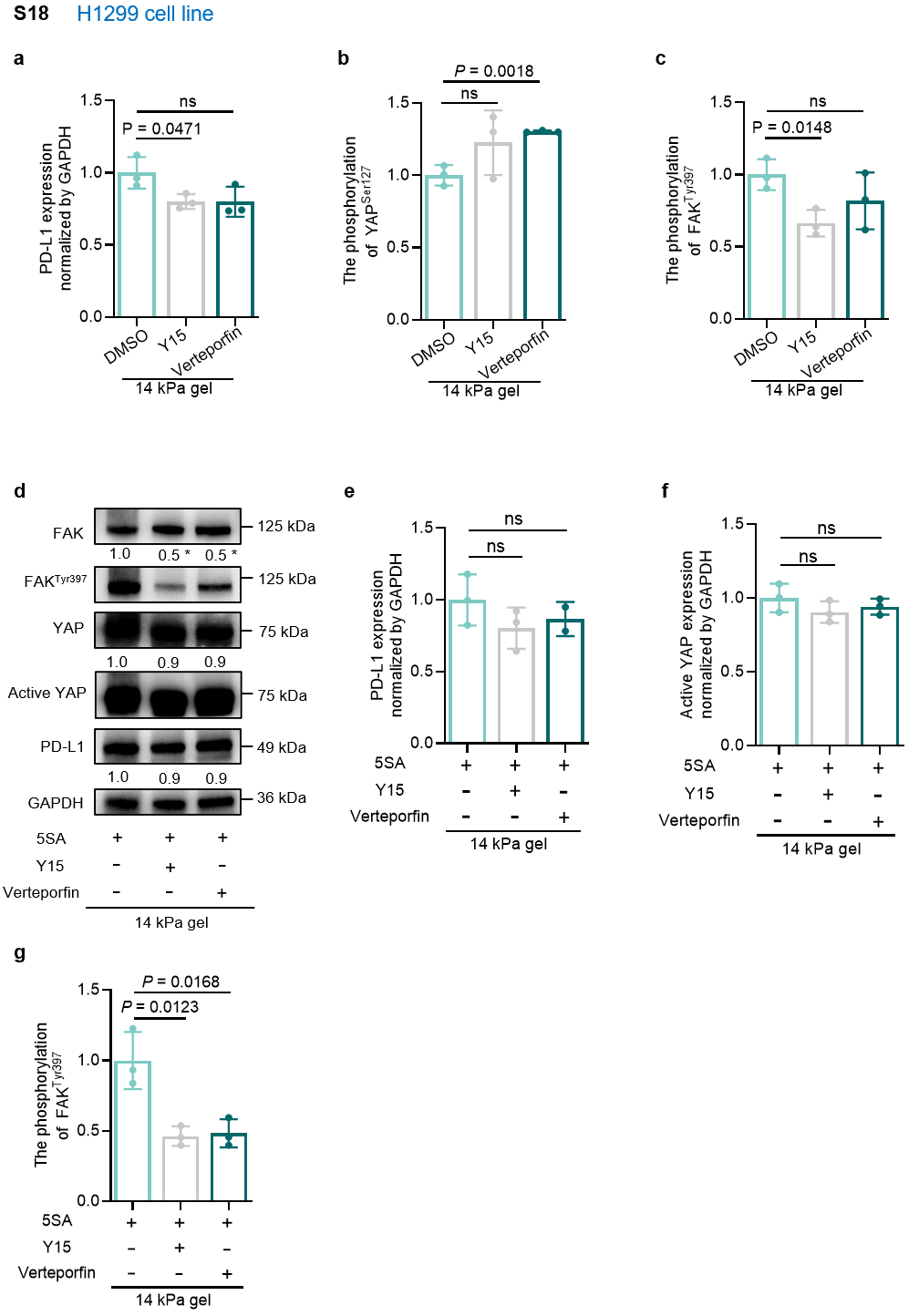


**Figure S18.** **Piezo1 activity regulates the expression of PD-L1 in cells cultured on optimal ECM stiffness through the FAK-YAP axis. a**, Quantification of the western blotting of the PD-L1 in H1299 cells treated with Y15 and Verteporfin on 14 kPa hydrogel. Protein expressions were normalized by GAPDH (N = 3). **b,** Quantification of phosphorylation of YAP^Ser127^ in H1299 cells treated with Y15 and Verteporfin on 14 kPa hydrogel. Degree of YAP^Ser127^ phosphorylation = gray value of YAP^Ser127^ / gray value of YAP (N = 3). **c,** Quantification of phosphorylation of FAK^Tyr397^ in H1299 cells treated with Y15 and Verteporfin on 14 kPa hydrogel. Degree of FAK^Tyr397^ phosphorylation = gray value of FAK^Tyr397^ / gray value of FAK (N = 3). **d**, The western blotting in YAP-5SA H1299 cells cultured on 14 kPa hydrogel with the treatment of Y15 and Verteporfin (N = 3). **e,** Quantification of the PD-L1 in YAP-5SA H1299 cells cultured on 14 kPa hydrogel with the treatment of Y15 and Verteporfin. Protein were expression normalized by GAPDH (N = 3). **f,** Quantification of the active YAP in YAP-5SA H1299 cells t cultured on 14 kPa hydrogel with the treatment of Y15 and Verteporfin. Protein were expression normalized by GAPDH (N = 3). **g,** Quantification of phosphorylation of FAK^Tyr397^ in YAP-5SA H1299 cells treated with Y15 and Verteporfin on 14 kPa hydrogel. Degree of FAK^Tyr397^ phosphorylation = gray value of FAK^Tyr397^ / gray value of FAK (N = 3). Data are compared by a two-tailed Student’s *t*-test (**a-c, e-g**). In (**a-c, e-g**), all data are shown as mean ± S.E.M. N, the number of independent experiments. 5SA, the plasmid of overexpression of constitutively active YAP; Verteporfin, YAP transcriptional function inhibitor; Y15, Phosphorylation inhibitor of FAK^Tyr397^.


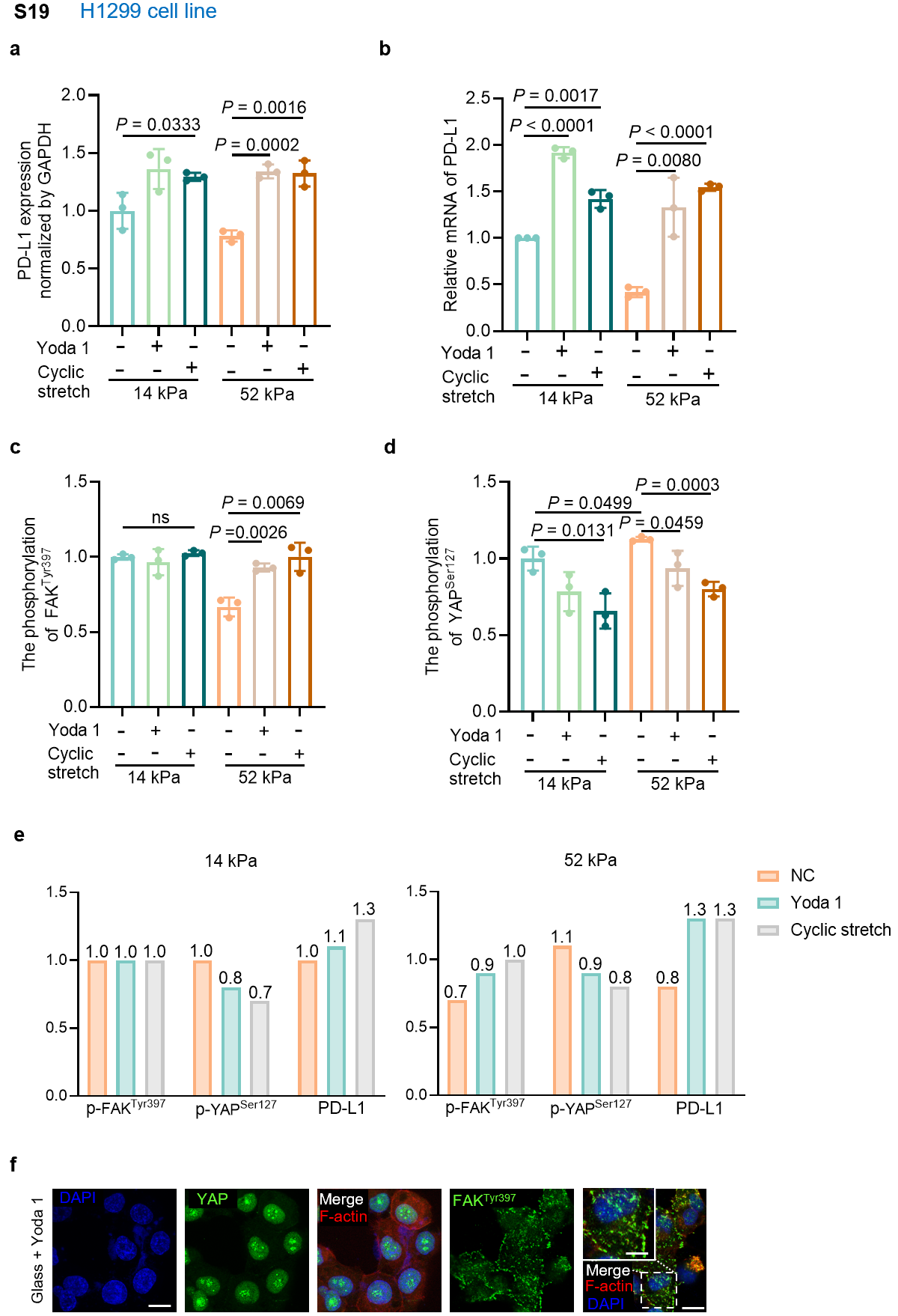


**Figure S19. Piezo1 activation overrides the optimal expression of PD-L1 by ECM stiffness via the FAK-YAP axis. a**, Quantification of PD-L1 in H1299 cells cultured on 14 kPa and 52 kPa hydrogels treated with Yoda 1 or cyclic stretch. Protein expression normalized by GAPDH (N = 3). **b**, The PD-L1 mRNA level in H1299 cells cultured on 14 kPa and 52 kPa hydrogels treated with Yoda 1 or cyclic stretch (N = 3). **c**, Quantification of the phosphorylation of FAK^Try397^ in H1299 cells cultured on 14 kPa and 52 kPa hydrogels treated with Yoda 1 or cyclic stretch. Degree of FAK^Tyr397^ phosphorylation = gray value of FAK^Tyr397^ / gray value of FAK (N = 3). **d**, Quantification of the phosphorylation of YAP^Ser127^ in H1299 cells cultured on 14 kPa and 52 kPa hydrogels treated with Yoda 1 or cyclic stretch. Degree of YAP^Ser127^ phosphorylation = gray value of YAP^Ser127^ / gray value of YAP (N = 3). **e,** The bar lot of the quantificated result of **Fig. 4b. f**, The immunofluorescence assay of YAP and FAK^Tyr397^ in H1299 cells cultured on glass treated by Yoda 1. The scale bars indicate 10 μm (enlarged image in the upper left corner) and 20 μm, respectively. The untreated control cells corresponding to **e** are presented in **Fig. 3d** (bottom row, control conditions on glass). Data are compared by a two-tailed Student’s *t*-test (**a-d**). In (**a-d**), all data are shown as mean ± S.E.M. N, the number of independent experiments.


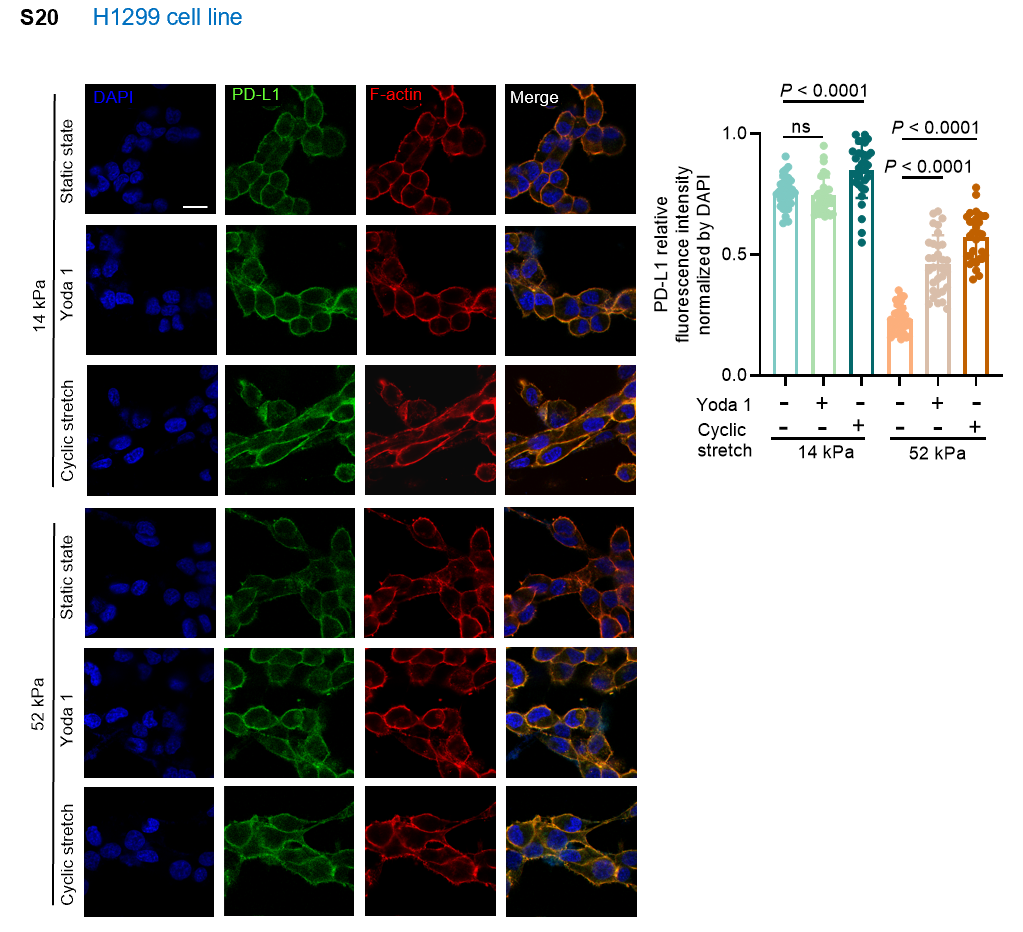


**Figure S20.** **Piezo1 activation overrides the optimal expression of PD-L1 by ECM stiffness in H1299 cells.** The immunofluorescence assay and the quantification of PD-L1 in H1299 cells cultured on 14 kPa and 52 kPa hydrogels treated with Yoda 1 or cyclic stretch. (N ≥ 5, n ≥ 30 cells). Data are compared by a two-tailed Student’s *t*-test. All data are shown as mean ± S.E.M. N, the number of independent experiments. n, the number of cells counted. ECM, extracellular matrix.


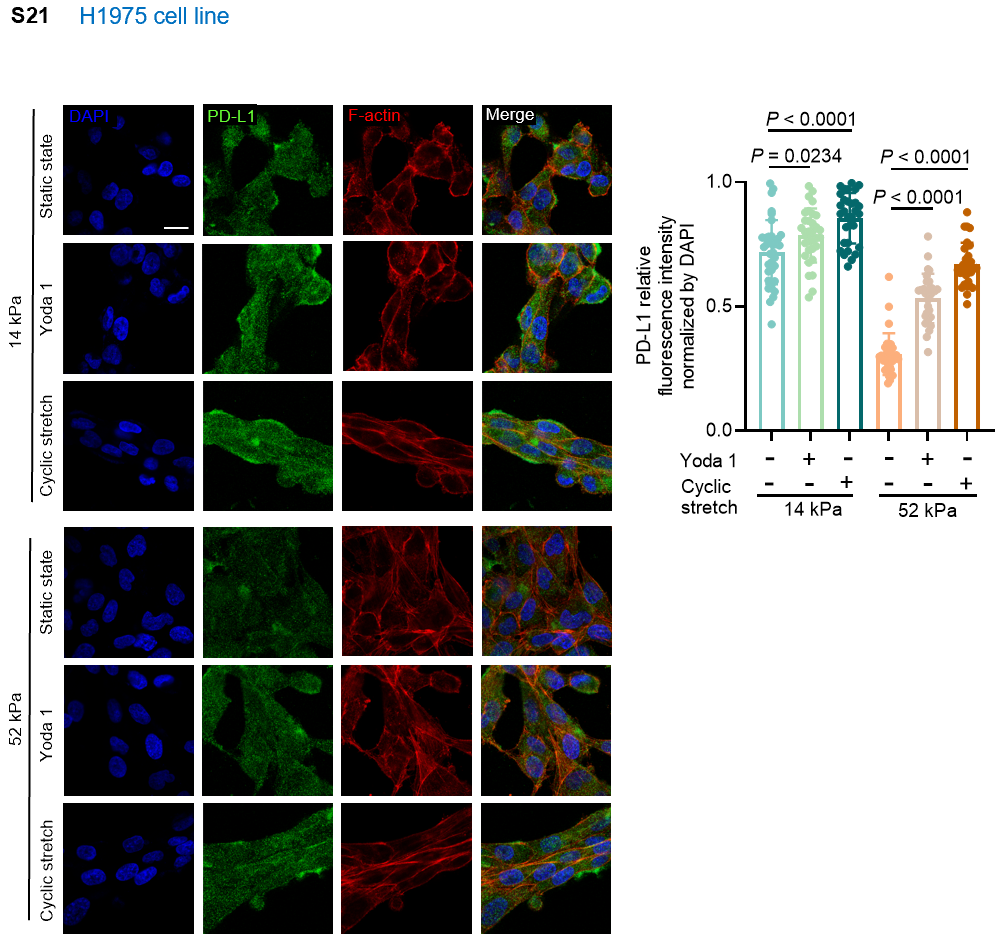


**Figure S21. Piezo1 activation overrides the optimal expression of PD-L1 by ECM stiffness in H1975 cells.** The immunofluorescence assay and the quantification of PD-L1 in H1975 cells cultured on 14 kPa and 52 kPa hydrogels treated with Yoda 1 or cyclic stretch. (N ≥ 5, n ≥ 30 cells). Data are compared by a two-tailed Student’s *t*-test. All data are shown as mean ± S.E.M. N, the number of independent experiments. n, the number of cells counted. ECM, extracellular matrix.


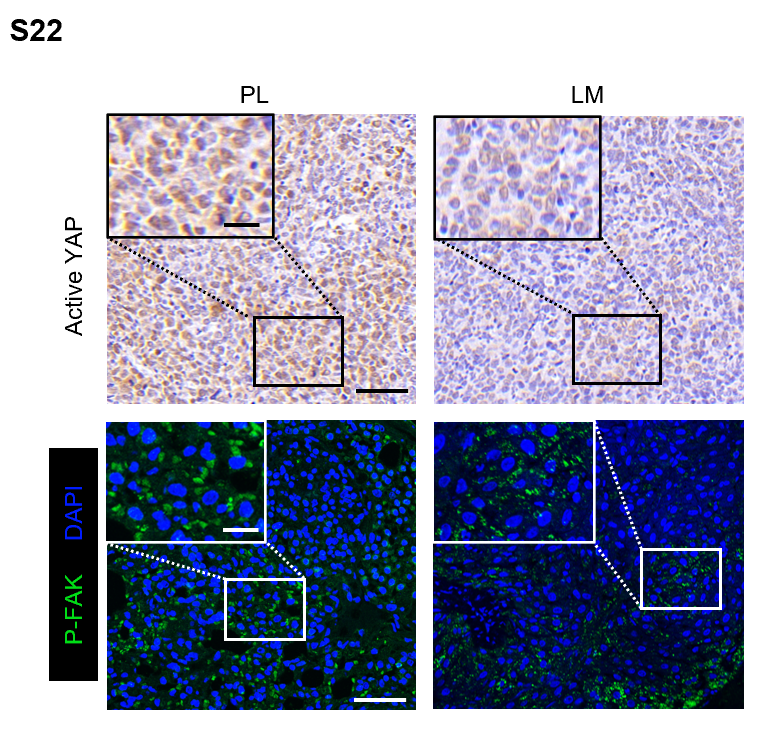


**Figure S22. The levels of active YAP and phosphorylation of FAK^Tyr397^ in PL compared to LM in murine models.** The scale bars of IHC staining indicate 20 μm (enlarged image in the upper left corner) and 60 μm, respectively. The scale bars of immunofluorescent images indicate 30 μm (enlarged image in the upper left corner) and 80 μm, respectively.


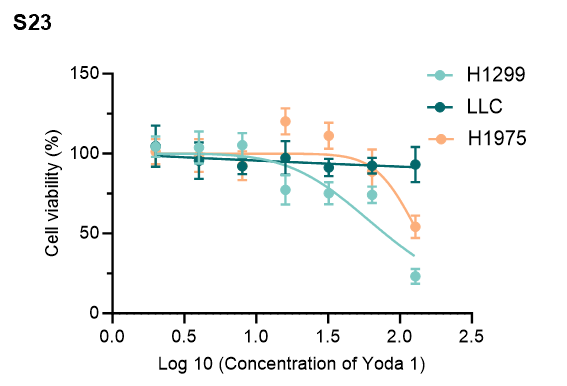


**Figure S23.** **Cytotoxicity test of Yoda 1 in cells. a**, MTT curves of different cell lines treated with Yoda 1 (N = 6). All data are shown as mean ± S.E.M. N, the number of independent experiments. Yoda 1, Piezo1-specific agonist.

**Table S1. Characteristics of lung cancer with liver metastasis patients receiving anti-PD-1 immunotherapy**

| **Characteristic** | **No. of patients (n=35)** |
| --- | --- |
| Median age, years (range) | 64 (41–75) |
| Sex, n (%) |  |
| Male | 31 (88.6) |
| Female | 4 (11.4) |
| Hypertension, n (%) |  |
| Yes | 5 (14.3) |
| No | 30 (85.7) |
| Smoking history, n (%) |  |
| Current/former | 22 (62.9) |
| Never | 13 (37.1) |
| Pathology, n (%) |  |
| Non-squamous | 23 (65.7) |
| Squamous | 12 (34.3) |
| Treatment line, n (%) |  |
| First | 11 (31.4) |
| Second | 14 (40) |
| Greater than or equal to third | 10 (28.6) |

**Table S2:** **The primer sequences and plasmid.**

| **Primer** | | |
| --- | --- | --- |
| *PD-L1* | GGACAAGCAGTGACCATCAAG | CCCAGAATTACCAAGTGAGTCCT |
| *CYR61* | CTCGCCTTAGTCGTCACCC | CGCCGAAGTTGCATTCCAG |
| *CTGF* | AAAAGTGCATCCGTACTCCCA | CCGTCGGTACATACTCCACAG |
| *GAPDH* | ACAACTTTGGTATCGTGGAAGG | GCCATCACGCCACAGTTTC |
| **Plasmid** | | |
| YAP-5SA plasmid | GENECHEM | Addgene, #33103 |
| Empty vector | GENECHEM | - |

**Table S3:** **Animal treatment protocol and schedule.**

|  | **On days 12, 18, 24** | **On days 15, 21, 27** |
| --- | --- | --- |
| Control group | 200 μl PBS | 20 μg per mice of isotype IgG (Bioxcell, BE0089, USA) |
| Yoda 1 group | 200 μg/kg of Yoda 1(HY-18723, MCE, USA) | 20 μg per mice of isotype IgG (Bioxcell, BE0089, USA) |
| Anti-PD-1 group | 200 μl PBS | 20 μg per mice of anti-PD-1 antibodies (Bioxcell, BE0273, USA) |
| Combo group | 200 μg/kg of Yoda 1(HY-18723, MCE, USA) | 20 μg per mice of anti-PD-1 antibodies (Bioxcell, BE0273, USA) |

*In vitro* experiments employed a concentration of 5 µM Yoda1 based on established studies demonstrating reliable Piezo1 activation with minimal toxicity in cultured cells[1, 2]. For in vivo experiments, we selected an intraperitoneal dosage of 200 µg/kg, aligning with previously validated doses that activate Piezo1 effectively while avoiding significant toxicity or systemic adverse reactions[3, 4].Every treatment was given by intraperitoneal injection.

**Table S4:** **The** **parameters of the mathematical model**

| **Symbol** | **Parameter** | **Value** |
| --- | --- | --- |
| *n_m_* | Total number of motors | 50 |
| $F_{stall}$ | Motor stall force | 20 pN |
| $V_{0}$ | Unloaded motor velocity | 120 nm/s |
| $F_{b}$ | Characteristic clutch rupture force | 2 pN |
| $K_{link}$ | Clutch spring constant | 1 pN |
| $K_{off0}$ | Clutch unload off rate | 0.1 s^-1^ |
| $k_{on}^{0}$ | Clutch on rate | 0.001 s^-1^ |
| $F_{cr}$ | Talin threshold force | 3 pN |
| $L_{0}$ | Initial length of actin | 2 μm |
| A | Cyclic stretch amplitude | 800 nm |
| $f_{r}$ | Cyclic stretch frequency | 0.125 Hz |

**Table S5:** **Equipments and softwores.**

| **Equipments and softwores** | **Brands / Version** |
| --- | --- |
| Nanowizard 4XP atomic force microscope | JPK Instruments |
| Inverted fluorescence microscope | Nikon ECLIPSE Ti2-E |
| Slice scanner | 3Dhistech Pannoramic Scan |
| Image J | Version 1.8.0 |
| BOSE ELECTROFORCE | BOSE 3200 |
| Clinx gel analysis | Clinx |
| Confocal scanning microscope | Olympus FV3000 |
| Olympus software | FV31S-SW |
| Immunofluorescence slice scanner | Pannoramic MIDI |
| Cell profiler^TM^ | Version 4.2.6 |

**Table S6:** **Regents.**

| **Regents** | **Cat.number** | **Dosage** |
| --- | --- | --- |
| Immunol Staining Blocking Buffer | Beyotime, P102 | - |
| Phosphate buffer saline | Servicebio, G002 | - |
| Immunohistochemistry kits | Cwbio, CW2069 | - |
| Hematoxylin dye | Beyotime, C0107 | - |
| PDMS base and crosslinker | Dow Corning, The Netherlands | 1:10 |
| Type-I collagen | Corning | 100 μg/ml |
| APS | A3678, Sigma | 1% |
| Tetramethylethylenedi-amine | T9281, Sigma | 0.1% v/v |
| Pierce™ Sulfo-SANPAH | Thermofisher, A35395 |  |
| HEPES | Aladdin | 0.5 M |
| Benzophenone | Aladdin | Dissolved in acetone (10 wt %) |
| Radio immunoprecipitation assay (RIPA) lysis buffer | Beyotime, P0013 | - |
| BCA Protein Assay Kit | Epizyme, ZJ102 | - |
| Loading buffer | Epizyme, LT101 | - |
| Blocking buffer | EpiZyme, PS108P | - |
| Universal Antibody Dilution Buffer | EpiZyme, PS119 | - |
| Light Chemiluminescence Kit | EpiZyme, SQ203 |  |
| Fluo-4 AM | Invitrogen | 5  μM |
| Hanks’ balanced salt solution (HBSS) | Solarbio | - |
| Total RNA Isolation Kit | Fastgen, RNAfast200 | - |
| cDNA Synthesis Kit | Novoprotein, E047 | - |
| SYBR High-Sensitivity qPCR SuperMix | Novoprotein, E099 | - |
| 4% Paraformaldehyde | Biosharp | - |
| Triton X-100 | Beyotime, P0096 | - |
| Rhodamine phalloidin | Solarbio, CA1680 | 1:200 |
| DAPI | MCE, HY-D0814 | 1:700 |
| E-trans | GENECHEM | - |
| Enzyme-linked immunosorbent assay kits | Cloud-Clone Corp, SEA371Hu | - |
| Four-color multiple fluorescent immunohistochemical staining kit | RS0035, ImmunoWay Biotechnology | - |

**Table S7:** **Drugs.**

| **Drugs** | **Brands** | **Dosage** |
| --- | --- | --- |
| Yoda 1 | MCE | 5 μM [1] |
| Y15 | MCE | 2 μM |
| GsMTx4 | MCE | 2.5 μM [5] |
| Veterporfin | MCE | 200 nM |

**Table S8:** **Antibodies.**

| **Antibody** | **Cat.number** | **Concentration** |
| --- | --- | --- |
| **Immunohistochemistry** | | |
| PD-L1 | Cell Signaling Technology, #13684 | 1:200 |
| CD8 alpha | Abcam, ab217344 | 1:1000 |
| Collagen I | Abcam, ab138492 | 1:1500 |
| CXCL10 | Proteintech, 10937-1-AP | 1:250 |
| active YAP | Abcam, ab205270 | 1:2000 |
| **Western blotting** | | |
| PD-L1 | Proteintech, 66248-Ig | 1:5000 |
| GAPDH | Proteintech, 60004-Ig | 1:20000 |
| YAP | Cell Signaling Technology, #14074 | 1:1000 |
| YAP^ser127^ | Cell Signaling Technology, #13008S | 1:1000 |
| Active YAP | Abcam, ab205270 | 1:1000 |
| FAK^Tyr397^ | Cell Signaling Technology, #3283 | 1:1000 |
| FAK | Cell Signaling Technology, #3285 | 1:1000 |
| HRP-labeled goat anti-mouse/rabbit secondary antibody | EpiZyme, LF102 / LF101 | 1:2000 |
| **Immunofluorescence staining** | | |
| Piezo1 | Proteintech, 15939-1-AP | 1:250 |
| active YAP | Abcam, ab205270 | 1:500 |
| FAK^Tyr397^ | Abcam, ab81298 | 5 μg/mL |
| Alexa Fluor® 488 (H + L) secondary antibody | Yeason, 34206ES60 | 1:500 |
| **Multiparametric immunofluorescence staining** | | |
| PD-L1 | Cell Signaling Technology, #13684 | 1:200 |
| CD8 alpha | Thermofisher, 14-0085-82 | 5 μg/mL |
| CXCL10 | Santa Cruz, sc-374092 | 1:250 |

**Table S9:** **Cell line, medium and animals.**

|  | **Source / Brands** |
| --- | --- |
| H1299 cell line | Culture Collection of the Chinese Academy of Sciences |
| H1975 cell line | Culture Collection of the Chinese Academy of Sciences |
| LLC | Culture Collection of the Chinese Academy of Sciences |
| C57BL/6 mice | Laboratory of Animal Research Center of Xi’an Jiaotong University |
| 1640 medium | Corning |
| 10% fetal bovine serum | Gibco |
| 100 U/mL penicillin and 100 μg/mL | Gibco |
| DMEM medium | Corning |

**SI** **Methods**

**1. Immunohistochemistry**

After fixation, deparaffinization and hydration, the samples were subjected to antigen retrieval. Then the samples were blocked and then added with primary antibodies for overnight incubation. The remaining steps were completed by immunohistochemistry kits following the manufacturer’s protocol. Cell nuclei were stained with hematoxylin dye. The number of immune cells is quantified by two fields of view per doner with an area of 356 μm*253 μm. The images were captured with an inverted fluorescence microscope and analyzed by ImageJ.

**2. Cell culture and treatment**

H1299 and H1975 cells were cultured in 1640 medium with 10% fetal bovine serum, 100 U/mL penicillin and 100 μg/mL streptomycin. LLC cells were cultured in DMEM medium with the same supplements. All cells were maintained in a humidified atmosphere with 5% CO_2_ at 37 °C. Cells were cultured for 24 hr before 24-hr treatment of drugs.

**3. Quantitative PCR**

Total RNA was isolated using RNA Isolation Kit and reverse transcribed using the cDNA Synthesis Kit. SYBR SuperMix was employed to evaluate the RNAs of interest. The mRNA levels were calculated with 2^–ΔΔCt^ method.

**4. Immunofluorescence staining and image analysis**

After fixation, permeabilization and blocking, cells were incubated with primary and secondary antibodies. Cell cytoskeleton and nucleus were stained by Rhodamine phalloidin and DAPI, respectively. The multiparametric staining was conducted with four-color multiple fluorescent immunohistochemical staining kit following the manufacturer’s protocol. The images were collected with Pannoramic MIDI and analyzed by Cell profiler software. The length of FAK^Tyr397^ FAs was assessed by using ImageJ as described previously [6]. The YAP n/c ratio was calculated following the protocol from literature [5].

**5. Plasmid and transfection**

H1299 cells were transfected with YAP-5SA plasmid and empty vector using transfection agent E-trans according to the manufacturer's instructions.

**6.** **RNA-sequencing (RNA-seq) and analysis**

RNA was harvested using RNA 6000 Nano LabChip Kit (Agilent, CA, USA, 5067-1511). 5 ug of total RNA was used for the construction of sequencing libraries. RNA libraries for RNA-seq were prepared using Dynabeads Oligo (dT) (Thermo Fisher, CA, USA) following manufacturer's protocols. A cDNA library constructed by the pooled RNA was sequenced run with Illumina NovaseqTM 6000 sequence platform.Using the Illumina paired-end RNA-seq approach, we sequenced the transcriptome, generating a total of millon 2 x 150 bp paired-end reads. All reads were further filtered by Cutadapt (<https://cutadapt.readthedocs.io/en/stable/,version:cutadapt-1.9>) to get high quality clean ones. We aligned reads of all samples to the Homo sapiens reference genome using HISAT2 (https://daehwankimlab.github.io/hisat2/,version:hisat2-2.0.4) package. The mapped reads were tassembled using StringTie (http://ccb.jhu.edu/software/stringtie/,version:stringtie-1.3.4d) with default parameters in each sample. Then, all transcriptomes from all samples were merged to reconstruct a comprehensive transcriptome using gffcompare software (<http://ccb.jhu.edu/software/stringtie/gffcompare.shtml,version:gffcompare-0.9.8>). The analysis of Genes differential expression was performed by DESeq2 software between two different groups (and by edgeR between two samples). The genes with the parameter of false discovery rate (FDR) below 0.05 and absolute fold change ≥ 2 were considered differentially expressed genes. Differentially expressed genes were then subjected to enrichment analysis of KEGG pathways. All bioinformatic analysis was performed using the OmicStudio tools at the website (<https://www.omicstudio.cn/tool>).

**7. Enzyme-linked immunosorbent assay (ELISA)**

The chemokine CXCL10 was tested using enzyme-linked immunosorbent assay kits following the manufacturer’s instructions.

**8. Mathematical model**

In this study, we have chosen to utilize a motor-clutch model to reveal the mechanism of these phenomena. While the standard motor-clutch model and its extensions have been utilized effectively to simulate the mechanical sensing of response matrix properties [7], the influence of the coupling between cyclic stretch and stiffness has not been accounted for. Consequently, we developed a motor-clutch model that incorporates cyclic stretch to investigate the effect of cyclic stretch on cell behavior with certain stiffness substrate. The motor-clutch model takes into account myosin motor proteins, actin filaments, integrin mediated molecular clutches, and elastic substrates. Actin filaments are pulled by myosin motor proteins, causing them to retrograde at a speed of $V_{0}$, even in the absence of external forces applied by cells. Additionally, the maximum pulling force that motors can provide is represented as $F_{stall}$ = *n_m_*$F_{m}$, where $F_{m}$ is the maximum force that each motor can exert. When the force $(F_{s})$ is transmitted through all connected clutches, the retrograde flow velocity of actin filaments, $V_{r}$, decreases linearly.

| $V_{r}\left( F_{s} \right)=V_{0}\left( 1-\frac{F_{s}}{F_{stall}} \right),$ | (1) |
| --- | --- |
| $F_{s}=K_{link}\sum_{i=1}^{N_{link}} (x_{i}-x_{s}),$ | (2) |

where $V_{0}$ is the base flow rate of actin filaments, $F_{stall}$ is the stall force of all myosin motors, $N_{link}$ is the number of engaged clutch bonds, $x_{i}$ is the elongation that spring *i*, and $x_{s}$ is the displacement of the substrate. In cells subjected to cyclic stretch, we apply forces at a fixed frequency ($f_{r}$) and amplitude (*A*) on actin filaments to simulate the conditions of cells undergoing cyclic stretch *in vivo*. The model combines the cyclic stretch velocity ($V_{c}$) with the retrograde flow velocity of actin filaments, altering the flow direction based on the frequency of stretching, thus modifying the retrograde flow speed.

| $V_{c}=A*f_{r},$ | (3) |
| --- | --- |
| $V_{e}=V_{e}+V_{c}$ | (4) |
| $F_{i}=K_{link}\left( x_{i}-x_{s} \right),$ | (5) |

The model represents the binding of clutches as parallel springs, with each spring having a spring constant $K_{link}$. According to the Bell model [7], the effective unbinding rate ($k_{off}$) of these individual clutch bonds is accelerated by the force ($F_{i}$) acting on each bond:

| $k_{off}=k_{off0}e^{\frac{F_{i}}{F_{b}}},$ | (6) |
| --- | --- |

where $F_{b}$ represents the rupture force of a clutch bond, and $k_{off0}$ is the initial unbinding rate of the clutches. Additionally, the binding and unbinding of clutches are highly dynamic co-occurring processes, and the rate of clutch binding $(k_{on}$) is regulated by the integrin density.

| $k_{on}=k_{on}^{0}C_{int},$ | (7) |
| --- | --- |

where$k_{on}^{0}$ is the initial binding rate of the clutches, and $C_{int}$ is the density of integrins on the cell membrane. On the other hand, as the tension experienced by each clutch bond increases, it triggers the unfolding of talin protein. Talin unfolding recruits integrins to enhance adhesion, so a threshold force is set in the simulation to trigger talin protein unfolding. When the threshold force ($F_{cr}$) offered by the clutches exceeds this threshold, $C_{int}$ continuously increases with the growth value ($D_{int}$). Conversely, when $F_{s}$ is less than the ($F_{cr}$), integrin density $C_{int}$ decreases with the value $D_{int}$.

Finally, simulation elucidated the mechanism behind alterations in cell behavior when subjected to cyclic stretch, as revealed by the adhesive length ($L_{A}$) and the YAP n/c ratio. Adhesive length is closely linked to both the total length of actin filaments ($L_{f}$) and the number of connections to integrins, whereas $L_{f}$ is intricately tied to the actual growth rate of actin filaments.

| $L_{f}=L_{0}+\left( V_{p}-V_{e} \right)*t$ | (8) |
| --- | --- |
| $L_{A}=L_{f}\left( \frac{N_{link}}{N_{tot}} \right)$ | (9) |

where $L_{0}$ is the initial length of actin; $V_{p}$ is the rate of actin aggregation; $N_{tot}$ is the total number of integrins binding sites. The parameters of the model are shown in **Table S3**

**References:**

[1] O.F. Harraz, N.R. Klug, A.J. Senatore, D.C. Hill-Eubanks, M.T. Nelson, Piezo1 Is a Mechanosensor Channel in Central Nervous System Capillaries, Circ Res, 130 (2022) 1531-1546.

[2] M. Li, X. Zhang, M. Wang, Y. Wang, J. Qian, X. Xing, Z. Wang, Y. You, K. Guo, J. Chen, D. Gao, Y. Zhao, L. Zhang, R. Chen, J. Cui, Z. Ren, Activation of Piezo1 contributes to matrix stiffness-induced angiogenesis in hepatocellular carcinoma, Cancer Commun (Lond), 42 (2022) 1162-1184.

[3] D. Choi, E. Park, R.P. Yu, M.N. Cooper, I.T. Cho, J. Choi, J. Yu, L. Zhao, J.I. Yum, J.S. Yu, B. Nakashima, S. Lee, Y.J. Seong, W. Jiao, C.J. Koh, P. Baluk, D.M. McDonald, S. Saraswathy, J.Y. Lee, N.L. Jeon, Z. Zhang, A.S. Huang, B. Zhou, A.K. Wong, Y.K. Hong, Piezo1-Regulated Mechanotransduction Controls Flow-Activated Lymphatic Expansion, Circ Res, 131 (2022) e2-e21.

[4] D. Choi, E. Park, E. Jung, B. Cha, S. Lee, J. Yu, P.M. Kim, S. Lee, Y.J. Hong, C.J. Koh, C.W. Cho, Y. Wu, N. Li Jeon, A.K. Wong, L. Shin, S.R. Kumar, I. Bermejo-Moreno, R.S. Srinivasan, I.T. Cho, Y.K. Hong, Piezo1 incorporates mechanical force signals into the genetic program that governs lymphatic valve development and maintenance, JCI Insight, 4 (2019).

[5] L. Niu, B. Cheng, G. Huang, K. Nan, S. Han, H. Ren, N. Liu, Y. Li, G.M. Genin, F. Xu, A positive mechanobiological feedback loop controls bistable switching of cardiac fibroblast phenotype, Cell Discov, 8 (2022) 84.

[6] Z. Kechagia, P. Sáez, M. Gómez-González, B. Canales, S. Viswanadha, M. Zamarbide, I. Andreu, T. Koorman, A.E.M. Beedle, A. Elosegui-Artola, P.W.B. Derksen, X. Trepat, M. Arroyo, P. Roca-Cusachs, The laminin-keratin link shields the nucleus from mechanical deformation and signalling, Nat Mater, (2023).

[7] C.E. Chan, D.J. Odde, Traction dynamics of filopodia on compliant substrates, Science, 322 (2008) 1687-1691.
